# Hepatitis C Virus Induces the Mitochondrial Translocation of Parkin and Subsequent Mitophagy

**DOI:** 10.1371/journal.ppat.1003285

**Published:** 2013-03-28

**Authors:** Seong-Jun Kim, Gulam H. Syed, Aleem Siddiqui

**Affiliations:** Division of Infectious Diseases, Department of Medicine, University of California, San Diego, La Jolla, California, United States of America; University of Southern California, United States of America

## Abstract

Hepatitis C Virus (HCV) induces intracellular events that trigger mitochondrial dysfunction and promote host metabolic alterations. Here, we investigated selective autophagic degradation of mitochondria (mitophagy) in HCV-infected cells. HCV infection stimulated Parkin and PINK1 gene expression, induced perinuclear clustering of mitochondria, and promoted mitochondrial translocation of Parkin, an initial event in mitophagy. Liver tissues from chronic HCV patients also exhibited notable levels of Parkin induction. Using multiple strategies involving confocal and electron microscopy, we demonstrated that HCV-infected cells display greater number of mitophagosomes and mitophagolysosomes compared to uninfected cells. HCV-induced mitophagy was evidenced by the colocalization of LC3 puncta with Parkin-associated mitochondria and lysosomes. Ultrastructural analysis by electron microscopy and immunoelectron microscopy also displayed engulfment of damaged mitochondria in double membrane vesicles in HCV-infected cells. The HCV-induced mitophagy occurred irrespective of genotypic differences. Silencing Parkin and PINK1 hindered HCV replication suggesting the functional relevance of mitophagy in HCV propagation. HCV-mediated decline of mitochondrial complex I enzyme activity was rescued by chemical inhibition of mitophagy or by Parkin silencing. Overall our results suggest that HCV induces Parkin-dependent mitophagy, which may have significant contribution in mitochondrial liver injury associated with chronic hepatitis C.

## Author Summary

Hepatitis C virus (HCV) infection alters host lipid metabolism. HCV-induced mitochondrial dysfunction may promote the metabolic alterations by affecting mitochondrial β-oxidation and oxidative phosphorylation. Dysfunctional mitochondria are detrimental to cell survival and require rapid clearance to sustain cell viability. Here, we investigated the effect of HCV gene expression in promoting selective autophagy of dysfunctional mitochondria, also termed mitophagy. HCV infection stimulated the gene expression of Parkin and PINK1, the two key mediators of mitophagy. Parkin stimulation was also observed in liver biopsies of chronic hepatitis C patients. HCV infection induced the perinuclear clustering of mitochondria and triggered Parkin translocation to mitochondria, a hallmark of mitophagy. Concomitant with the mitochondrial translocation of Parkin, we observed ubiquitination of Parkin and its substrates in HCV-infected cells. We also demonstrate the formation of mitophagosomes and their subsequent delivery to lysosomes in HCV-infected cells. Silencing both Parkin and PINK1 hindered HCV replication, suggesting the functional significance of mitophagy in HCV life cycle. Furthermore, we demonstrate that Parkin-dependent mitophagy is directly associated with HCV-mediated decline in oxidative phosphorylation. Our results implicate the functional significance of Parkin and mitophagy in the persistence of HCV infection and mitochondrial injury commonly seen in patients with chronic hepatitis C.

## Introduction

Hepatitis C virus (HCV) infection most often leads to chronic hepatitis, which can progress to steatosis, fibrosis, cirrhosis, and hepatocellular carcinoma [1]. HCV RNA genome encodes a polyprotein, which includes; core, E1, E2, p7, NS2, NS3, NS4A, NS4B, NS5A, and NS5B [2]. Viral RNA replicates in the endoplasmic reticulum (ER)-derived modified membranous structures and is subsequently assembled on lipid droplets [3], [4]. Most of the viral proteins are either associated with or tethered to the ER [3]. These associations and relevant activities overburden the ER and induce an ER stress response exhibited by the unfolded protein response (UPR) [5].

ER stress response is a potent inducer of autophagy [6]. Several reports have described that HCV gene expression perturbs the autophagic pathway to induce bulk autophagy [7]–[17]. Several reports have highlighted the functional role of autophagic machinery in various steps of HCV life cycle (viral replication, translation, and propagation) [7]–[17]. HCV-induced UPR and autophagy have also been functionally linked to inactivation of innate antiviral response [10], [18]. Interestingly, recent reports hint that HCV-induced autophagosomal membrane may serve as platform for HCV replication [11], [19]. However, further evidence is required in support of this notion. Overall these results are consistent with the notion that viruses in general either induce or suppress autophagy to benefit the infectious process [20], [21]. Although recent studies describe the involvement of bulk autophagy in various steps of HCV lifecycle, our understanding of the biological significance and the precise role of autophagy in HCV lifecycle are still rudimentary.

HCV infection is associated with physiological insults like ER stress, oxidative stress, ROS accumulation, and mitochondrial Ca^2+^ overload that can trigger collapse of mitochondrial transmembrane potential (ΔΨm) and subsequent mitochondrial dysfunction [5], [22]–[26]. Quality control of the dysfunctional mitochondrial is essential to sustain the bioenergetic efficiency and to prevent the initiation of mitochondria-mediated intrinsic cell death signaling cascade [27].

In humans, loss of function and mutations in genes encoding Parkin and PINK1 are linked to autosomal recessive form of Parkinson's disease [28], [29]. Recent advances have unraveled their functional role in selective autophagic elimination of damaged mitochondria, termed as mitophagy [30]. Parkin is an E3 ubiquitin ligase, which is normally localized in the cytoplasm. However, upon mitochondrial stress, it is rapidly recruited to the damaged mitochondria [28], [31], [32]. PINK1, a mitochondrial Ser/Thr kinase, recruits Parkin on depolarized mitochondria and interacts with mitochondrial outer membrane complex to regulate Parkin translocation and activation [30], [33].

In this study, we investigated the involvement of mitophagy in HCV-infected cells. Our results demonstrate that HCV induces Parkin-dependent mitophagy. HCV infection dramatically triggered Parkin translocation to mitochondria, which was convincingly demonstrated both by confocal microscopy and subcellular fractionation of highly pure mitochondria. HCV stimulated Parkin and PINK1 gene expression at transcriptional level. Electron microscopy of HCV-infected cells displayed engulfment of damaged mitochondria in double membrane vesicles and subsequent fusion of these mitochondria-containing vesicles with the lysosome. These results were further strengthened by immunoelectron microscopy. In HCV-infected cells, Parkin associated with mitochondria was ubiquitinated and promoted the degradation of its mitochondrial substrates. Further, our results showed that silencing Parkin and PINK1 hindered HCV replication suggesting the functional relevance of mitophagy in HCV replication. HCV-induced mitophagy was also directly associated with HCV-mediated decline in mitochondrial complex I enzyme activity. Overall our results implicate that HCV-induced mitophagy is physiologically relevant in maintenance of cellular homeostasis, persistence of HCV infection, and liver disease pathogenesis.

## Results

### HCV induces mitochondrial perinuclear clustering and Parkin translocation to mitochondria

**Figure 1:**
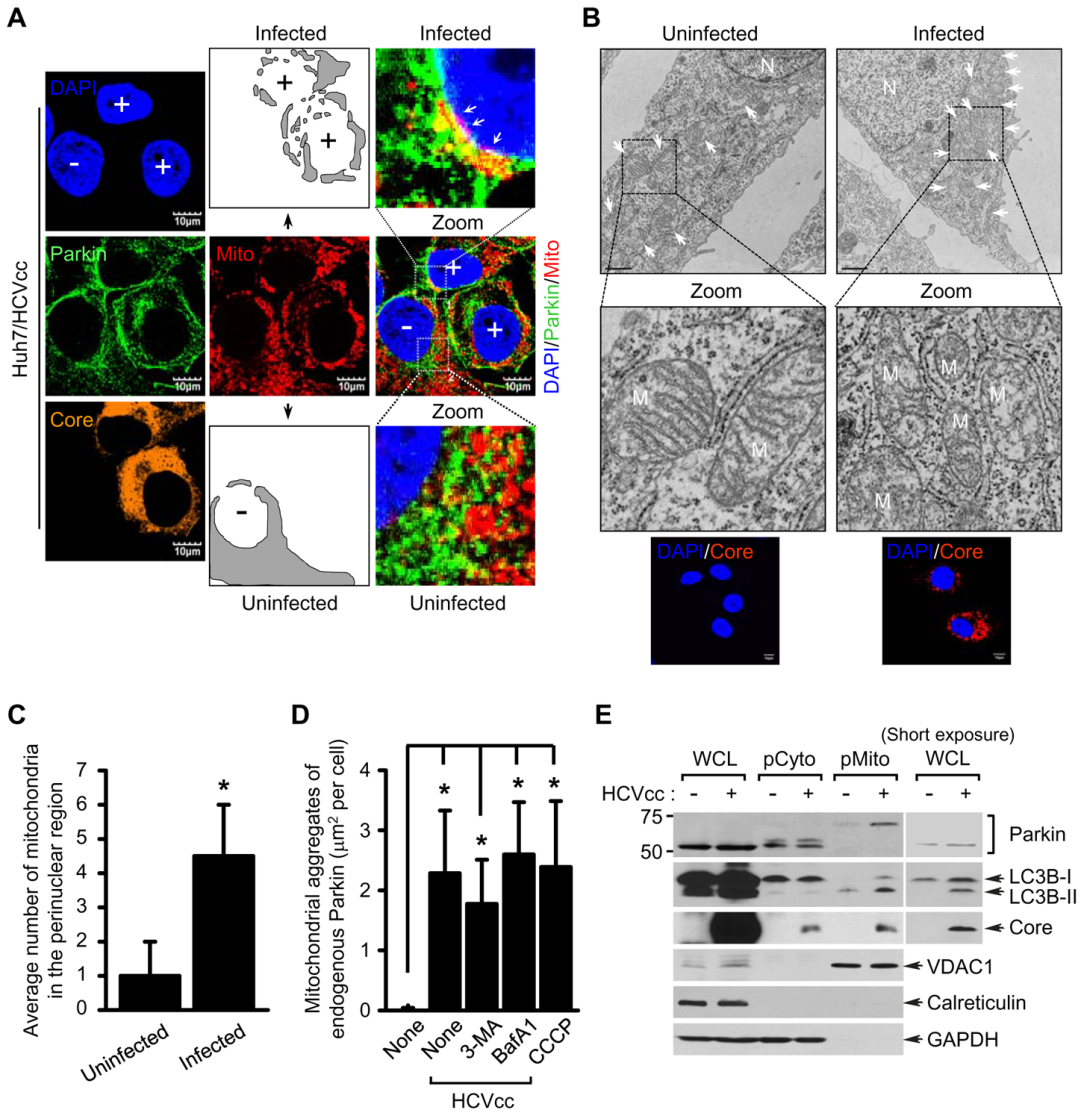
HCV infection induces perinuclear clustering of mitochondria and Parkin translocation to mitochondria. (**A**) Representative confocal images showing perinuclear clustering of mitochondria and Parkin aggregates on the mitochondria in Huh7 cells infected with HCVcc. At 3 days post-infection, cells prestained with MitoTracker (Mito, red) were immunostained with anti-Parkin (green) and HCV core (orange) antibodies. Nuclei were stained with DAPI (blue). Infected (+) and uninfected cells (−) are marked. The illustrated images display typical distribution of mitochondria in uninfected cells and altered distribution of mitochondria in the perinuclear region of infected cells (gray). In the zoomed images, the white arrows indicate accumulation of endogenous Parkin recruited to the mitochondria in HCV-infected cells (yellow). (**B**) Ultrastructure of HCV-infected cells showing perinuclear clustering of damaged mitochondria. Control naïve Huh7 cells (left) and stable cells harboring HCV full-length replicon FLR-JFH1 (right) were examined by electron microscopy. In the zoomed images, typical ultrastructure of mitochondria in naïve cells and ultrastructural abnormalities of mitochondria in HCV replicon cells are shown. Organelle mark: N, nucleus; M and white arrow, mitochondria. Scale bar = 1 µM. Fluorescent images (below) indicate the expression of HCV core protein in HCV replicon cells. Cells were immunostained with anti-HCV core antibody (red). Nuclei were stained with DAPI (blue). (**C**) Quantification of the number of mitochondria in the perinuclear region (mean ± SEM; n≥5 cells; *p<0.05). (**D**) Quantification of fluorescence intensity of Parkin aggregates on the mitochondria in CCCP-treated (see **Figure S1**) or HCV-infected cells (**A**) and those treated with 3-MA or BafA1, respectively (see **Figure 4A**) (mean ± SEM; n = 10 cells; *p<0.01). P values were calculated by using an unpaired Student's t-test. (**E**) Western blot analysis showing endogenous Parkin recruitment to mitochondria in HCV-infected cells. Huh7 cells were infected with HCVcc. At 5 days post-infection, pure cytoplasm and mitochondria fractions were isolated by ultracentrifugation as described in Materials and Methods. Cellular fractions of HCV-infected cells were analyzed by immunoblotting using antibodies specific for the indicated proteins. Fractions: whole cell lysates, WCL; purified cytoplasm, pCyto; purified mitochondria, pMito. Organelle markers: VDAC1, mitochondria; Calreticulin, ER; GAPDH, cytoplasm.

Parkin translocation to mitochondria is a well-characterized event that triggers the induction of mitophagy [30]. To investigate HCV-induced mitophagy, we assessed mitochondrial translocation of Parkin in cell culture-derived HCV Jc1 strain (hereafter referred to as HCVcc) infected human hepatoma Huh7 cells by immunofluorescence imaging [34]. In these images, a significant perinuclear clustering of mitochondria in HCV-infected cells was observed (Figure 1A). To further strengthen this observation, we conducted electron microscopy (EM) using Huh7 cells harboring HCV full-length replicon (FLR-JFH1) [35] (Figure 1B) or those infected with HCVcc (Figure S2). EM analysis of these cells revealed prominent clustering of mitochondria in the perinuclear regions of HCV-infected cells, displaying a dramatic loss of mitochondrial cristae (Figures 1B, 1C, S1A and S1C). In contrast, in the uninfected cells typical cytoplasmic distribution of mitochondria with intact cristae was observed (Figures 1A and B). Oxidative stress has been shown to induce the spheroid formation of damaged mitochondria [36]. Using immunoelectron microscopy, we noted similar perinuclear clustering of impaired mitochondria as well as mitochondrial spheroid formation (Figures S1C and S1D).

Cyanide m-cholorophenyl hydrazone (CCCP) triggers mitochondrial translocation of Parkin [32]. Mitochondrial translocation and aggregation of Parkin was observed in CCCP-treated Huh-7 cells (Figure S2) as well was in HCV-infected (Figure 1A). Quantitative analysis of mitochondrial translocation of Parkin is presented in Figure 1D. HCV-induced Parkin translocation was further investigated by immunoelectron microscopy as described in Fig. S11A. HCV infected cells were stained with antibodies specific to TOM20, HCV E2, and Parkin, respectively and treated with secondary antibodies conjugated with 18-nm, 12-nm and 6-nm gold particles, respectively. As can be seen, Parkin is localized to damaged mitochondria with notable loss of cristae (Figure S11A).

**Figure 2:**
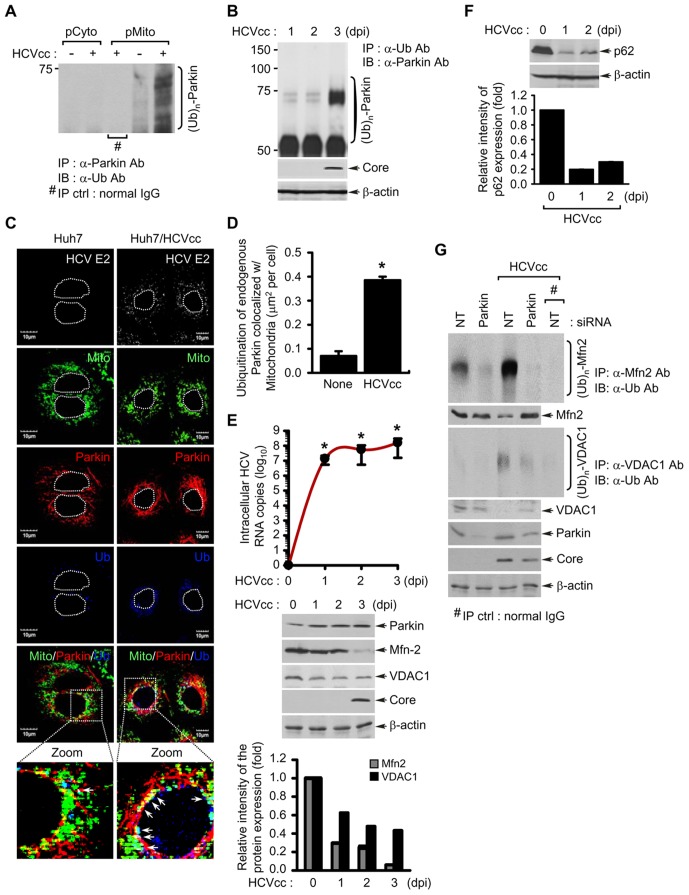
HCV infection induces the ubiquitination of Parkin and its mitochondrial substrates Mfn2 and VDAC1, and autophagy-associated factor p62. (**A**) Parkin ubiquitination in the purified mitochondria isolated from HCV-infected cells. Parkin protein in pCyto and pMito fractions, respectively, was immunoprecipitated by anti-Parkin antibody, followed by immunoblotting with anti-ubiquitin (Ub) antibody. Normal rabbit IgG was used as a negative control for immunoprecipitation (IP). (**B, E, and F**) At the indicated time points post-infection, WCL were extracted from HCV-infected Huh7 cells. (**B**) Parkin ubiquitination in WCL extracted from HCV-infected cells. All ubiquitinated proteins were immunoprecipitated with anti-Ub antibody and analyzed by immunoblotting with anti-Parkin antibody. HCV infection was verified by immunoblotting with anti-HCV core antibody. β-actin was used as an internal loading control. (**C**) Confocal microscopy showing Parkin ubiquitination in HCV-infected cells. HCV-infected cells prestained with MitoTracker (Mito) were immunostained with anti-Parkin and ubiquitin (Ub) antibodies. In the zoomed images, the arrows (white spots) indicate the merge of Mito (green), Parkin (red), and Ub (blue). Nuclei are demarcated with white dot circles. Fluorescence image of HCV E2 verifies HCV-infected cells (light gray). (**D**) Quantitative analysis of the ubiquitination of endogenous Parkin colocalized with mitochondria in the panels (**C**) (mean ± SEM; n≥10 cells; *p<0.05). (**E**) Western blot analyses of Mfn2 and VDAC1 expression, mitochondrial substrate of Parkin, in HCV-infected cells. Intracellular HCV RNA levels were analyzed by real-time qRT-PCR, as described in Materials and Methods (mean ± SD; n = 3; *p<0.01). WCL extracted from HCV-infected cells were analyzed by immunoblotting with anti-Parkin, Mfn2, and VDAC1 antibodies, respectively. HCV infection was verified by immunoblotting with anti-HCV core antibody. β-actin was used as an internal loading control. (**F**) Western blot analysis of p62 expression, the autophagy-associated factor, in HCV-infected cells. WCL extracted from HCV-infected cells were analyzed by immunoblotting with anti-p62 antibody. β-actin was used as an internal loading control. (**E and F**) The relative intensity of Mfn2, VDAC1, and p62 expression normalized to β-actin was analyzed by ImageJ. (**G**) Parkin-mediated ubiquitination of Mfn2 and VDAC1 in HCV-infected cells. The ubiquitinated Mfn2 and VDAC1 proteins were analyzed by immunoprecipitation with anti-Mfn2 and VDAC1 antibodies, respectively, followed by immunoblotting with anti-Ub antibody. The protein expression levels of Parkin were analyzed by immunoblotting with anti-Parkin antibody. HCV infection was verified by immunoblotting with anti-HCV core antibody. β-actin was used as an internal loading control. Normal mouse IgG was used as a negative control for immunoprecipitation (IP). (**D** and **E**) P values were calculated by using an unpaired Student's t-test.

Next, we utilized purified mitochondrial and cytosolic fractions of HCV-infected and uninfected Huh7 cells to examine Parkin translocation. Western blot analysis of these fractions is presented in Figure 1E. Parkin was highly enriched and predominantly ubiquitinated in the pure mitochondrial fraction (pMito) of HCV-infected cells (Figure 1E). This result was further substantiated by immunoprecipitation of Parkin in pMito fraction with anti-Parkin antibody followed by subsequent immunoblotting with anti-ubiquitin antibody (Figure 2A). Further, we carried out immunoprecipitation with anti-ubiquitin antibody using whole cell lysates of HCV-infected cells followed by subsequent immunoblotting with anti-Parkin antibody. As shown in Figures 2A and 2B, significant levels of ubiquitinated endogenous Parkin were detected during HCV infection. Substantial levels of Parkin ubiquitination on mitochondria were also observed in HCV-infected cells by immunofluorescence analysis (Figure 2C). Quantitative analysis of endogenous Parkin ubiquitination on mitochondria is presented in Figure 2D. Parkin is an E3 ubiquitin ligase, which exerts its E3 ubiquitin ligase activity by ubiquitinating itself and other mitochondrial target proteins [30], [31]. A mitochondrial outer membrane protein mitofusin 2 (Mfn2) and voltage-dependent anion-selective channel 1 (VDAC1) are known substrates of Parkin [30], [37]. Indeed, both Mfn2 and VDAC1 levels were reduced concomitant to increased Parkin levels during HCV infection (Figure 2E). To examine whether HCV infection induces Parkin-mediated ubiquitination of Mfn2 and VDAC1, we performed immunoprecipitation of HCV infected cells with anti-Mfn2 and VDAC1 antibodies followed by subsequent immunoblotting with anti-ubiquitin antibody (Figure 2G). This analysis revealed that both Mfn2 and VDAC1 were significantly ubiquitinated (Figures S4 and S5). This is further strengthened by confocal images of HCV infected cells showing enhanced ubiquitination of Mfn2 and VDAC1 (Figures S4 and S5). Furthermore, these increased ubiquitination of Mfn2 and VDAC1 were attenuated by Parkin silencing. Thus, these results indicate that HCV infection enhances Parkin-mediated ubiquitination of its substrates (Figure 2G).

p62/Sequestosome 1 (p62) has been previously shown to cooperate with Parkin during perinuclear clustering of mitochondria and required for Parkin-mediated mitophagy [38], [39]. Thus, we investigated the degradation of p62 during HCV infection. As shown in Figure 2F, Western blot analysis of HCV infected cells exhibited a decrease in p62 level. We also observed that HCV infection enhances the interaction of p62 and Parkin on mitochondria and results in subsequent increase of p62 ubiquitination in HCV-infected cells (Figures S6 and S7).

Interestingly, a majority of LC3B protein enriched in the mitochondrial fraction of HCV-infected cells was lipidated, which indicates the formation of phagophore involving mitochondria, an early event of mitophagy [30] (Figure 1E). Consistent with previous reports, HCV core is also enriched in mitochondria (Figure 1E) [23], [40]–[43]. We also examined mitochondrial translocation of Parkin in Huh7 cells harboring HCV 2a full-length (FLR-JFH1) or subgenomic replicon (SGR-JFH1) and Huh7.5.1 cells harboring HCV 1b subgenomic replicon (BM4–5 Feo) [35], [44], [45] (Figure S3). Parkin recruitment to the perinuclear mitochondrial clusters was uniformly observed in full-length and subgenomic replicons of genotype 2a and 1b subgenomic replicon cells, suggesting that this phenomenon is independent of the differences in HCV genotypes.

### HCV stimulates Parkin and PINK1 gene expression

**Figure 3:**
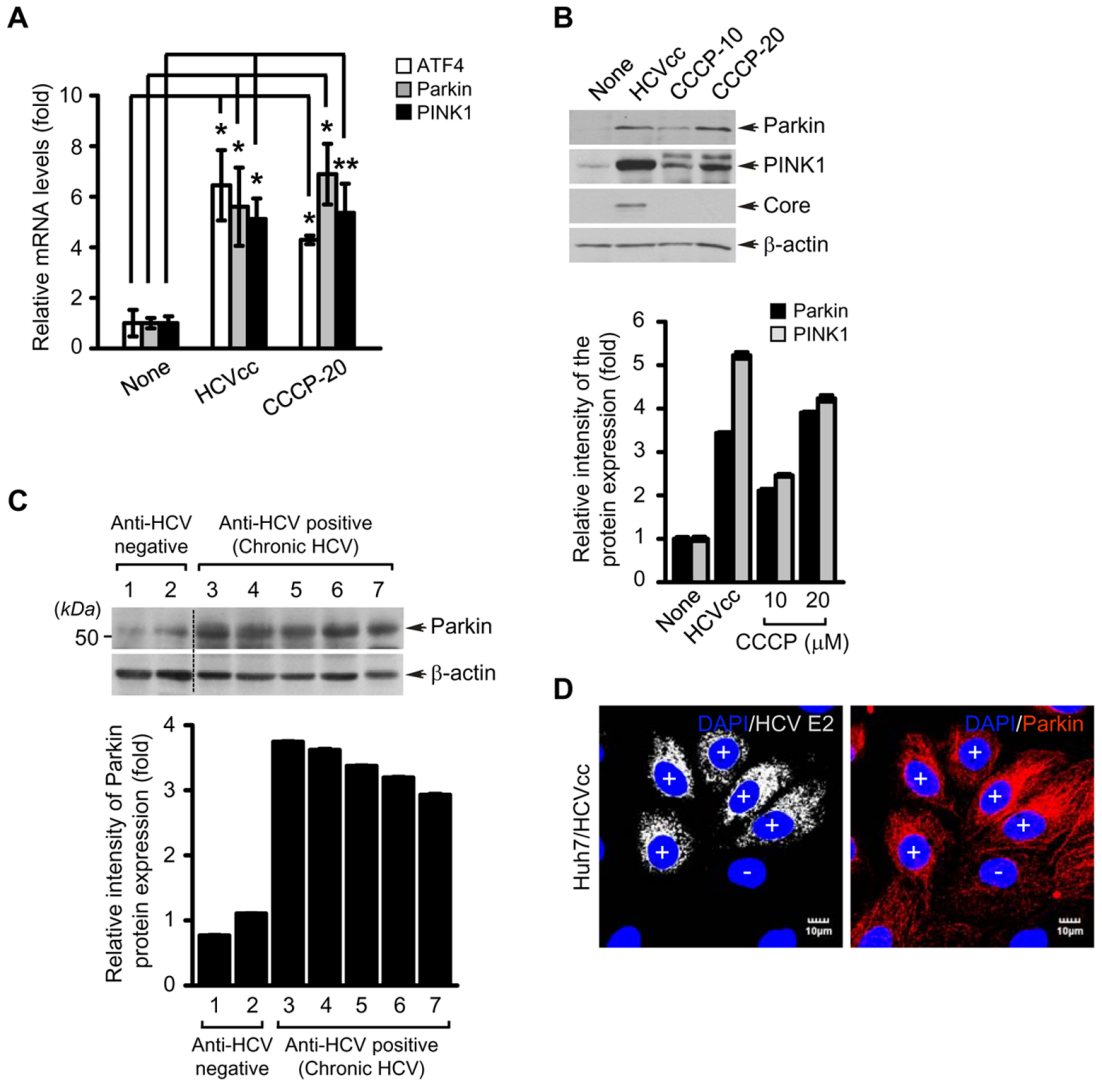
HCV infection stimulates ATF4, Parkin, and PINK1 gene expression. (**A and B**) Quantitative analyses of ATF4, Parkin, and PINK1 gene expression in HCV-infected cells. Huh7 cells infected with HCVcc for 5 days or treated with CCCP (10 or 20 µM) for 24 h were used for analysis of ATF4, Parkin, and PINK1 gene expression. (**A**) Intracellular mRNA levels of ATF4, Parkin, and PINK1 were analyzed by real-time qRT-PCR. GAPDH was used to normalize changes in Parkin and PINK1 gene expression (mean ± SD; n = 3; *p<0.01, **p<0.05). P values were calculated by using an unpaired Student's t-test. (**B**) The protein expression levels of Parkin and PINK1 were analyzed by immunoblotting with anti-Parkin and PINK1 antibodies. The expression of HCV core protein was analyzed by immunoblotting with anti-HCV core antibody. β-actin was used as an internal loading control. (**C**) Western blot analysis of Parkin expression in liver biopsies of chronic hepatitis C patients. Samples 1 and 2 represent liver biopsies of HCV-negative patients and samples 3–7 represent HCV-positive chronic hepatitis patients. (**B and C**) The relative intensity of Parkin and PINK1 expression normalized to β-actin analyzed by ImageJ. (**D**) Confocal microscopy showing Parkin overexpression in HCV-infected cells. Huh7 cells infected with HCVcc were immunostained with anti-Parkin antibody (red). Fluorescence image of HCV E2 verifies HCV-infected cells (light gray). Nuclei were stained with DAPI (blue). Infected (+) and uninfected cells (−) are marked.

We then examined if HCV infection stimulates the expression of Parkin and PINK1, two key regulators of mitophagy. As shown in Figures 3A and B, HCV infection stimulated both the mRNA and protein levels of Parkin and PINK1. CCCP-treatment also stimulated Parkin and PINK1 gene expression, in agreement with previous reports [46], [47] (Figure 3B). Further, confocal microscopy also revealed that Parkin protein expression is stimulated in HCV-infected cells compared to uninfected cells (Figure 3D). ATF4 is the transcription factor that is activated during UPR, which promotes Parkin upregulation both by ER and mitochondrial stress [46]. Increased ATF4 expression has also been reported in HCV chronic liver tissues [48]. In the present analysis, we observed ATF4 transcriptional stimulation in HCV infected cells, in agreement with previous reports [49], [50] (Figure 3A). Parkin expression was also analyzed in liver biopsies of chronic hepatitis C patients. We observed a consistent increase in Parkin protein level in the liver biopsies of all the chronic hepatitis C patients compared to non-hepatitis C donors (Figure 3C).

### HCV promotes Parkin-dependent mitophagosome formation

**Figure 4:**
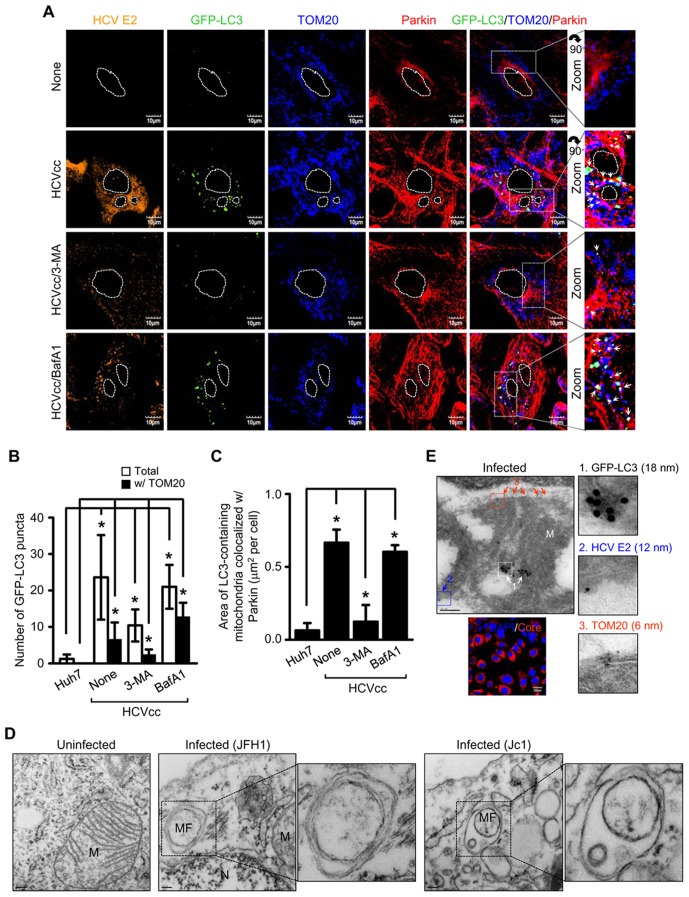
HCV induces Parkin-mediated mitophagosome formation. Confocal microscopy showing mitophagosome formation associated with Parkin in HCV-infected cells. (**A**) Huh7 cells transiently expressing GFP-LC3 protein were infected with HCVcc in the absence or presence of 3-MA (10 mM) and BafA1 (100 nM), respectively, for 12 h before fixation. At 3 day post-infection, cells were immunostained with antibodies against HCV E2 (orange), TOM20 (blue), and Parkin (red). In the zoom images, the arrows (white puncta) indicate GFP-LC3 puncta (green) colocalized with TOM20 and Parkin. Nuclei are demarcated with white dot circles. (**B**) Quantification of the number of GFP-LC3 puncta colocalized with TOM20 in the panel (**A**) (mean ± SEM; n≥10 cells; *p<0.01). (**C**) Quantitative analysis of the area of GFP-LC3 puncta (white) representing merge of GFP-LC3 puncta, TOM20, and Parkin in the panel (**A**) (mean ± SEM; n≥10 cells; *p<0.001). (**B** and **C**) P values were calculated by using an unpaired Student's t-test. (**D**) Ultrastructure of HCV-infected cells showing the formation of mitophagosome. Control naïve Huh7 cells (uninfected), HCV FLR-JFH1 replicon cells, and Huh7 cells infected with HCVcc (Jc1) were examined by electron microscopy. In the zoomed image, the formation of mitophagosome in HCV-infected cells is shown. Organelle mark: N, nucleus; MF, mitophagosome formation. Scale bar = 0.2 µM (uninfected); 0.1 µM (infected). (**E**) Immunoelectron microscopic analysis showing the formation of mitophagosome in HCV-infected cells. Huh7 cells infected with HCVcc were stained by triple immunogold labeling method, as described in Materials and Methods. The immunogold particles of GFP-LC3 (white arrow, 18 nm), HCV E2 (blue arrow, 12 nm), and TOM20 (red arrow, 6 nm) are shown. Fluorescence image of HCV core identifies HCV-infected cells (red). Nuclei were stained with DAPI (blue). Scale bar = 0.2 µM.

To analyze HCV-induced mitophagosome formation, Huh7 cells ectopically expressing GFP-LC3 were infected with HCVcc and analyzed by immunofluorescence imaging. We observed numerous GFP-LC3 puncta in HCV-infected cells (Figure 4A). Analysis of mitophagosome formation by merge of GFP-LC3 puncta with TOM20, a mitochondrial marker, revealed significantly higher number of mitophagosomes in HCV-infected cells compared to uninfected cells (Figures 4A and B). We further observed the colocalization of Parkin with mitophagosomes, which signifies the role of Parkin in mediating mitophagosome formation (Figure 4A). To strengthen these observations, we established stable cell line expressing Parkin-specific shRNA (P-KD). Using P-KD cells ectopically expressing GFP-LC3, we observed only a few GFP-LC3 puncta in HCV infected cells that do not localize to mitochondria, signifying the role of Parkin in mitophagy (Figure S8). Whereas, in non-targeting shRNA (NT-KD) stable cells infected with HCV, mitophagosome formation was observed (Figure S8). 3-methlyadenine (3-MA) inhibits the phagophore formation, whereas Bafilomycin A1 (BafA1), a specific inhibitor of vacuolar-type H^+^-ATPase, blocks the downstream step of fusion between the autophagosomes and lysosomes, resulting in the accumulation of undegraded autophagosomes (Figure S9) [51], [52]. 3-MA treatment of HCV-infected cells significantly reduced not only the number of total GFP-LC3 puncta but also mitophagosome-specific GFP-LC3 puncta, whereas BafA1 treatment resulted in their accumulation (Figures 4A and B). Quantitative analysis of GFP-LC3 puncta associated with TOM20 and Parkin is summarized in Figure 4C. The proteolytic cleavage and transient lipidation of LC3 (conversion from LC3-I to LC3-II) with phosphatidylethanolamine is essential for phagophore expansion [53]. In correlation with earlier studies, conversion ratio of LC3-II/LC3-I with anti-LC3B antibody is increased in HCV-infected cells compared to uninfected cells (Figure 1E, see the short exposure panel). To confirm mitophagosome formation in HCV-infected cells, we performed electron microscopy using Huh7 cells harboring HCV full-length replicon (FLR-JFH1) or those infected with HCVcc. Ultrastructural analysis of HCV-infected cells revealed damaged mitochondria, displaying traces of cristae or no cristae (empty) (Figures 1B, S1, and 4D). The empty mitochondria or mitochondria with traces of cristae are engulfed by double membrane vesicles in HCV-infected cells (Figure 4D). In contrast, uninfected Huh7 cells displayed normal mitochondrial morphology with typical cristae (Figure 4D). Formation of mitophagososmes was further analyzed by immunoelectron microscopy. HCV infected cells were labeled with antibodies specific to GFP-LC3, HCV E2, and TOM20, respectively and subsequently treated with secondary antibodies conjugated with 18-nm, 12-nm and 6-nm gold particles, respectively. As shown in Figures 4E and S11B, GFP-LC3 colocalizes on the damaged mitochondria in HCV-infected cells, thus confirming HCV-induced formation of mitophagosome. Together, these results strongly demonstrate that HCV-infected cells display higher incidence of Parkin-mediated mitophagosome formation.

### HCV induces the formation of mitophagolysosome

**Figure 5:**
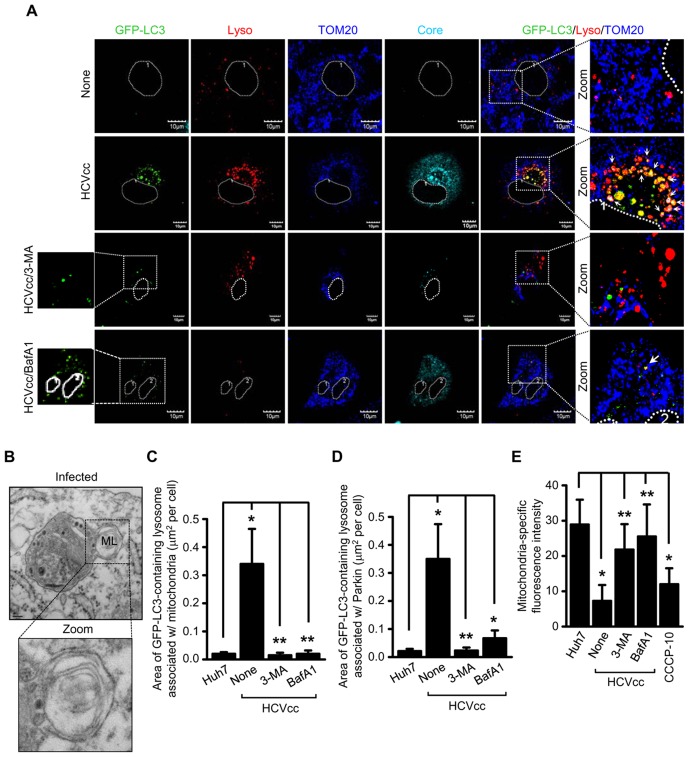
HCV induces complete mitophagolysosome formation. Confocal microscopy showing the formation of mitophagolysosome in HCV-infected cells. (**A**) Huh7 cells transiently expressing GFP-LC3 protein were infected with HCVcc in the absence or presence of 3-MA (10 mM) and BafA1 (100 nM), respectively, for 12 h before fixation. At 3 days post-infection, cells prestained with LysoTracker (red) were immunostained with antibodies against TOM20 (blue) and HCV core (cyan). Nuclei are demarcated with white dot circles. In the zoomed images, the arrows indicate the colocalization of GFP-LC3 puncta (green), lysosome, and mitochondria in HCV-infected cells (white puncta). (**B**) Ultrastructure of HCV-infected cells showing the formation of mitophagolysosome. HCV FLR-JFH1 replicon cells were examined by electron microscopy. In the zoomed image, the formation of mitophagolysosome in HCV-infected cells is shown. Organelle marker: ML, mitophagolysosome. Scale bar = 0.1 µM. (**C and D**) Quantitative analyses of the colocalization of GFP-LC3 containing lysosomes with mitochondria (**C**) or Parkin (**D**) in the panels (**A**) and **Figure S5** (mean ± SEM; n≥10 cells; *p<0.001, **p<0.05). (**E**) ImageJ quantitative analysis of the number of mitochondria (mean ± SEM; n≥10 cells; *p<0.001, **p<0.05). P values were calculated by using an unpaired Student's t-test.

Next, we determined the formation of mitophagolysosome or the fusion of mitophagosome with lysosomes. HCV-infected cells transiently expressing GFP-LC3 were stained with LysoTracker and/or lysosomal-associated membrane protein 2 (LAMP2). Immunofluorescence analysis revealed that HCV-infected cells displayed several distinct lysosomes containing mitochondria-associated with GFP-LC3 in comparison to uninfected cells (Figure 5A). The analysis of colocalization between lysosomes (immunostained with LAMP2) and Parkin-associated GFP-LC3 puncta in HCV-infected cells revealed several distinct lysosomes that were positive for Parkin and GFP-LC3 (Figure S10). In the presence of either 3-MA or BafA1, such colocalizations were severely reduced (Figures 5A and S10). In support of these observations, we performed electron microscopy using HCV full-length replicon-bearing Huh7 cells. HCV-infected cells revealed fusion of lysosomes with double membrane vesicles containing damaged mitochondria (Figure 5B). Next, we conducted immunoelectron microscopy of HCVcc infected Huh7 cells. Cells were labeled with antibodies specific to LAMP1, HCV E2, and Parkin, respectively and treated with secondary antibodies conjugated with immunogold 18-nm, 12-nm, and 6-nm gold particles, respectively. As shown in Figure S11C, fusion of LAMP1-positive lysosome and damaged mitochondria containing Parkin was observed in HCV-infected cells. These observations confirm and support results of confocal immunofluorescence imaging that HCV infection induces the formation of mitophagolysosome. Quantitative analyses of colocalization between lysosomes and GFP-LC3 puncta-associated with mitochondria or Parkin are represented in Figures 5C and D. Quantitative analysis of mitochondria-specific fluorescence intensity revealed significant reduction of mitochondria in HCV-infected cells that was restored to normal levels by treatment with both 3-MA and BafA1 (Figure 5E). As previously described, CCCP treatment also resulted in a decline in the number of mitochondria [54] (Figure 5E). Together, these results clearly suggest that mitophagosomes formed in HCV-infected cells subsequently fuse with lysosomes leading to the formation of mitophagolysosomes.

### Parkin and PINK1 affects HCV replication

**Figure 6:**
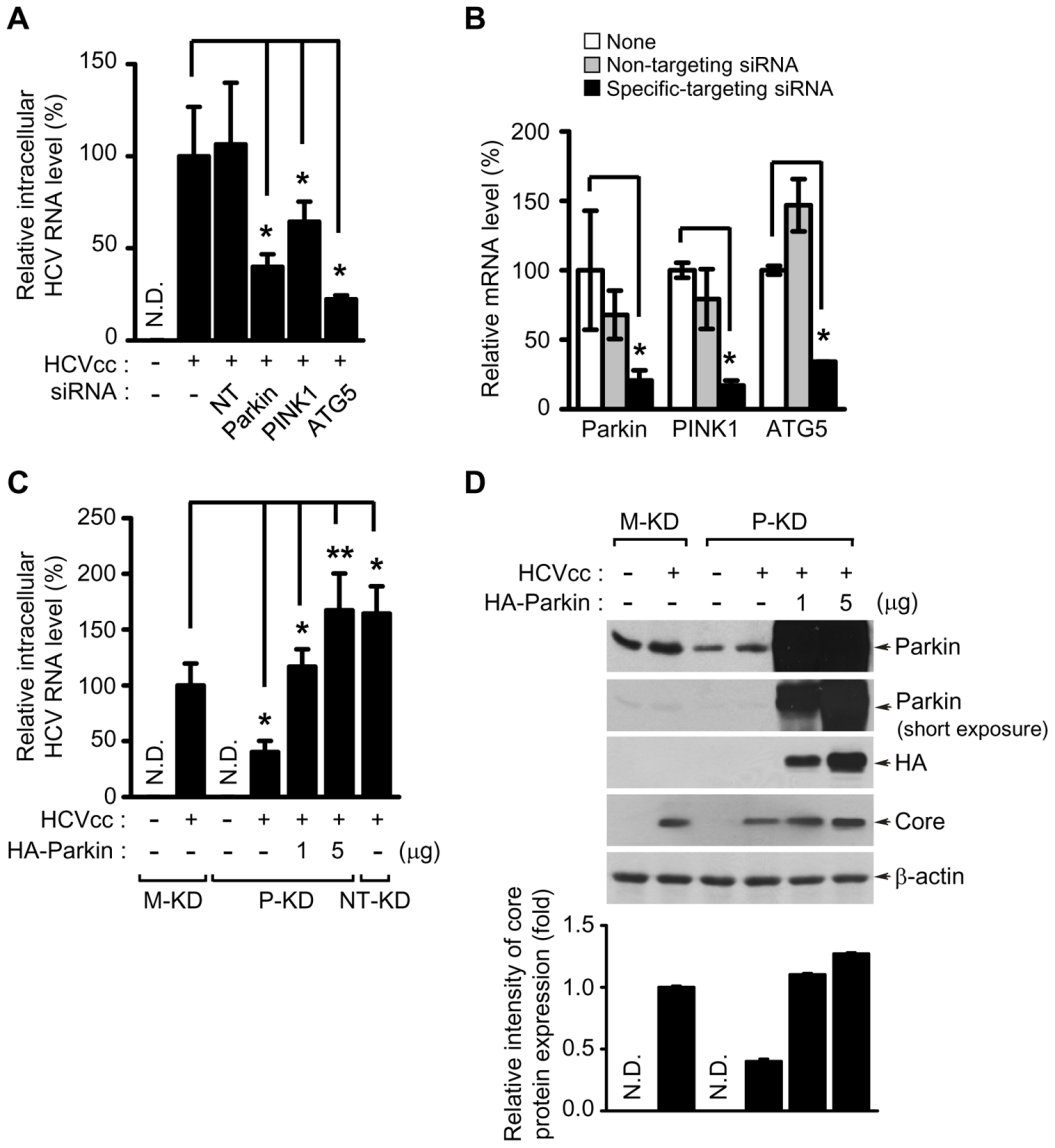
Parkin and PINK1 affects HCV replication. (**A** and **B**) Inhibitory effect of Parkin, PINKI, and ATG5 silencing on HCV replication. Huh7 cells transfected with Non-Targeting (NT) or gene-specific siRNA pools targeting Parkin, PINK1, and ATG5 genes, respectively, were infected with HCVcc. At day 3 post-infection, the levels of HCV RNA (**A**) and targeted gene mRNA (**B**) were analyzed by real-time qRT-PCR, as described in Materials and Methods (mean ± SD; n = 3; *p<0.01). GAPDH was used to normalize changes in Parkin, PINK1, and ATG5 gene expression. (**C** and **D**) Rescue of HCV RNA replication by Parkin overexpression. Huh7 cells stably expressing mock vector (M-KD), Non-target-shRNA (NT-KD), and Parkin-shRNA (P-KD), respectively, were infected with HCVcc. P-KD cells were further transfected with two different concentration of the plasmid DNA encoding wild-type Parkin for 2 days before harvest. At 3 days post-infection, intracellular HCV RNA levels were analyzed by real-time qRT-PCR. GAPDH was used to normalize changes in HCV RNA expression (**C**) (mean ± SD; n = 3; *p<0.01, **p<0.05). (**D**) The expression levels of Parkin and HCV core protein were analyzed by immunoblotting with anti-Parkin and HCV core antibodies. Ectopic expression level of HA-tagged wild-type Parkin was detected by immunoblotting with anti-HA antibody. β-actin was used as an internal loading control. P values were calculated by using an unpaired Student's t-test. The relative intensity of HCV core expression normalized to β-actin was analyzed by ImageJ.

To evaluate the functional role of Parkin and PINK1 in the HCV infectious process, we employed the siRNA strategy to silence the gene expression of Parkin and PINKI. We first verified silencing of Parkin and PINK1 gene expression by qRT-PCR analysis (Figure 6B). Analysis of HCV RNA replication in the presence of respective gene-specific siRNAs shows that Parkin and PINK1 silencing effectively inhibited HCV replication (Figure 6A). Atg5, a key regulator of phagophore elongation, has been previously shown to be involved in HCV replication [10], [55]. Atg5 silencing by siRNA inhibited HCV RNA replication in the cells infected with HCVcc (Figures 6A and B).

To further study silencing effect of Parkin on HCV replication, we analyzed HCV replication using P-KD cells infected with HCVcc. Consequently, HCV RNA replication levels declined in P-KD cells compared to NT-KD cells (Figure 6C). Parkin-specific shRNA did not affect cellular viability as determined by the resazurin-based cytotoxicity (Figures S12). We then performed a Parkin rescue experiment in HCV-infected P-KD cells. Dose-dependent ectopic expression of shRNA-resistant Parkin rescued the inhibition effect of Parkin silencing on HCV RNA replication (Figure 6C). In correlation with the rescue of HCV RNA replication by ectopic expression of shRNA-resistant Parkin, we observed corresponding stimulation of HCV core expression (Figure 6D). These results directly implicate a functional role of Parkin and PINK1 in HCV replication.

### HCV infection affects mitochondrial oxidative phosphorylation in the context of Parkin

**Figure 7:**
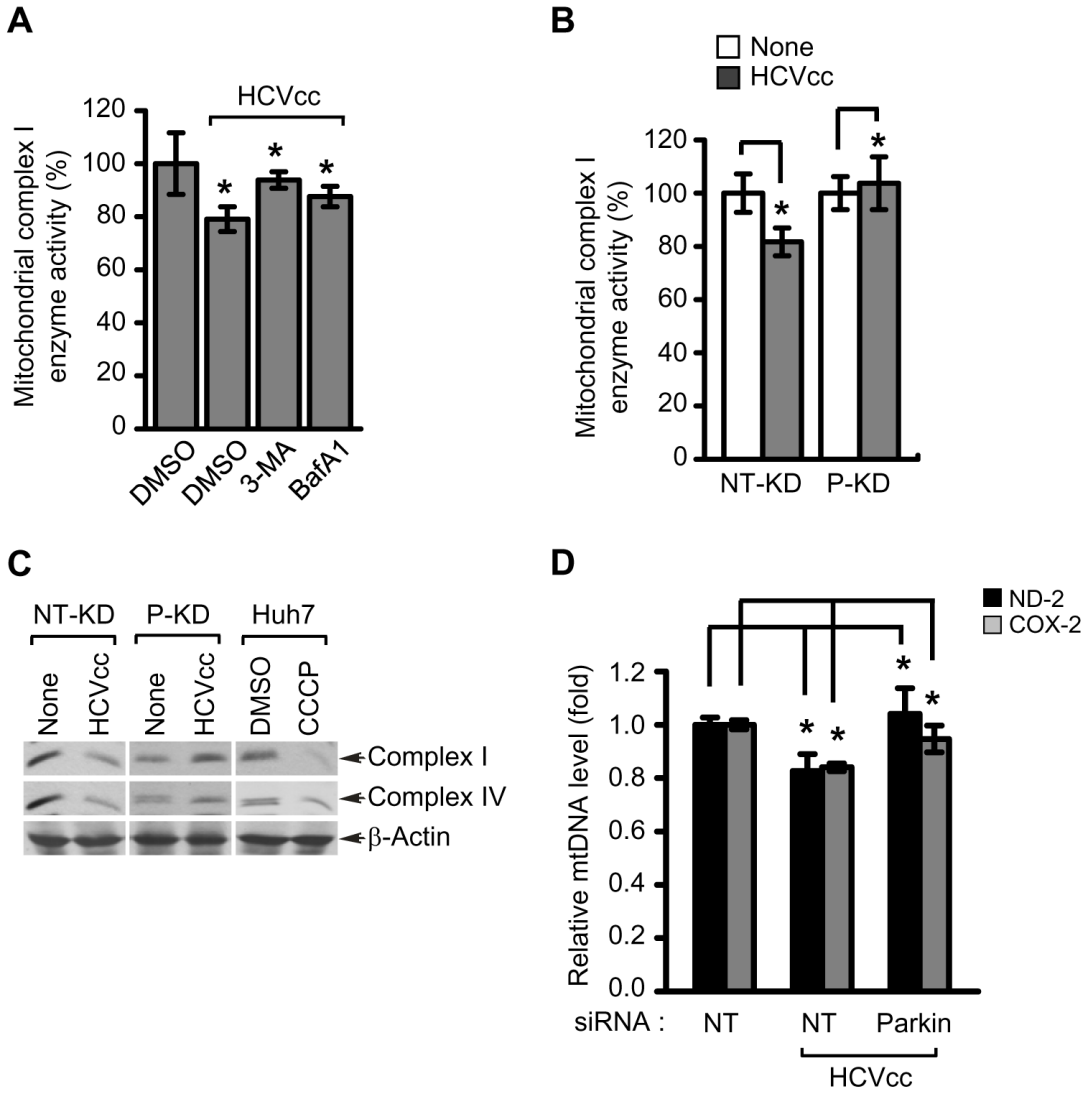
Mitochondrial functions altered by HCV infection are associated with Parkin-mediated mitophagy. (**A**) Rescue effect of 3-MA and BafA1 on reduction of mitochondrial complex I enzyme activity caused by HCV infection. Huh7 cells infected with HCVcc were treated with 3-MA (10 mM) or BafA1 (100 nM) for 12 h before harvest. At 3 days post-infection, the activity of mitochondrial complex I enzyme was measured according to manufacturer's instructions (mean ± SD; n = 3; *p<0.01). (**B**) Rescue effect of Parkin knockdown on reduction of mitochondrial complex I enzyme activity caused by HCV infection. NT-KD and P-KD cells infected with HCVcc were harvested on day 3 post-infection and used for analysis of the activity of mitochondrial complex I enzyme (mean ± SD; n = 3; *p<0.05). (**C**) Western blot analysis of mitochondrial respiratory chain complex enzyme expression. NT-KD and P-KD cells were infected with HCVcc and at 3 days post-infection, the expression levels of complex I and IV enzyme were analyzed by immunoblotting with anti-Hu total OxPhos complex antibody. Huh7 cells treated with CCCP (10 µM) for 12 h were also analyzed as a control. β-actin was used as an internal loading control. P values were calculated by using an unpaired Student's t-test. (**D**) Effect of Parkin silencing on depletion of mitochondrial DNA caused by HCV infection. Huh7 cells transfected with NT or Parkin-specific siRNA pools were infected with HCVcc. At day 3 post-infection, mitochondrial DNA levels of ND-2 and COX-2 were analyzed by real-time qPCR. GAPDH was used to normalize changes in ND-2 and COX-2 gene expression (mean ± SD; n = 3; *p<0.01). (**A**, **B**, and **D**) P values were calculated by using an unpaired Student's t-test.

Previous reports have shown that HCV infection affects mitochondrial oxidative phosphorylation [23], [24], [56]. Parkin is also shown to influence mitochondrial oxidative phosphorylation by promoting the degradation of mitochondrial outer membrane proteins [30]. Hence, we speculated that HCV triggered decline in oxidative phosphorylation is a consequence of Parkin-mediated mitophagy triggered by HCV infection. We measured the mitochondrial respiratory chain complex I enzyme (complex 1) activity in HCV-infected cells in the presence and absence of 3-MA and BafA1. HCV infection resulted in the reduction of mitochondrial complex I activity, which was restored by treatment of both 3-MA and BafA1 (Figure 7A). Consistent with previous reports [23], [24], we observed that HCV infection leads to about 20–25% decline in mitochondrial complex 1 activity (Figure 7A). To determine the effect of Parkin on HCV-mediated decline of mitochondrial complex I activity, P-KD and NT-KD cells were infected with HCVcc and mitochondrial complex I activity determined. HCV infection decreased mitochondrial complex I activity in NT-KD cells, whereas P-KD cells failed to show any decrease in mitochondrial complex I activity (Figure 7B). HCV has been previously shown to downregulate the expression of mitochondrial complex I and IV enzymes [57]. To further strengthen these results, we pursued the analysis of expression levels of complex I and IV enzymes in HCV infected cells in the context of Parkin. Consistent with previous reports, HCV infection affected the expression levels of both mitochondrial complex I and IV enzymes in NT-KD cells [57]. In contrast, the expression levels of mitochondrial complex I and IV enzymes in P-KD cells were unaffected by HCV infection (Figure 7C). Changes in the expression of mitochondrial complex I and IV enzymes were also detected in CCCP-treated Huh7 cells. It has been previously shown that chronic HCV infection results in the depletion of mitochondrial DNA (mtDNA) in the liver [58]. Here, we examined the effect of Parkin silencing on HCV-induced mtDNA depletion. Analysis of mitochondrial NADH dehydrogenase-2 (ND-2) and cytochrome c oxidase-2 (COX-2) in the presence of specific siRNA for Parkin shows that Parkin silencing effectively blocked HCV-induced decline of mtDNA levels (Figure 7D). Taken together, these results indicate that HCV-induced mitophagy is functionally associated with HCV-mediated impairment of oxidative phosphorylation and depletion of mitochondria.

## Discussion

Autophagy involves clearance of protein aggregates, damaged mitochondria, peroxisosmes and bacteria and viruses [53]. A growing body of literature on autophagy implicates its role in cellular homeostasis, innate immunity, defense mechanisms against lethal entities and in maintenance of persistent viral infections [21]. Pathogens have developed strategies to usurp autophagic pathways and hijack the machinery to favor viral proliferation and persistent infection [20]. Several reports described the HCV-induced events of bulk autophagy and have linked this pathway to aiding viral proliferation [9], [10], [12], [13], [17], [55]. The requirement of autophagy was emphatically shown by silencing key components of autophagy (Atg5, Atg7, Beclin-1, Atg8) respectively, and observing a decline in viral translation and replication process [9], [10], [12], [13], [17], [55]. Here, for the first time, we demonstrate that HCV induces organelle selective autophagic degradation of mitochondria (mitophagy). This was shown by marked translocation of Parkin to mitochondria (Figures 1, S3, and S11) in HCV-infected cells. Parkin translocation to mitochondria is considered a hallmark of mitophagy [30]. We also observed a significant stimulation of Parkin expression in both HCV-infected Huh7 cells and liver tissues samples obtained from chronic HCV patients (Figure 3). Similarly, HCV also stimulated the expression of PINK1, another key component of mitophagy involved in recruitment of Parkin to damaged mitochondria and its subsequent activation.

Diverse independent reports suggest the importance of autophagy machinery in multiple steps of HCV life cycle such as, translation, replication and secretion raising considerable controversy in depicting the precise role of autophagy in HCV infection [7]–[17]. In addition, some previous reports also claim that HCV induces incomplete bulk autophagy and prevents the fusion of autophagosomes with lysosomes [13]. Ke and Chen showed that HCV promotes complete autophagy, which culminates in the formation of autophagolysosome and that this event is crucial for viral replication [10]. Here, we also show the formation of mitophagosomes and mitophagolysosomes as evidenced by the presence of lipidated LC3B in pure mitochondrial fraction and the colocalization of GFP-LC3 puncta with Parkin loaded mitochondria and the subsequent fusion/colocalization of these mitophagosomes with lysosomes in HCV-infected cells (Figures 1, 4, 5, and S10, S11). To further reinforce our observation we performed ultrastructural and immunogold-labeled electron microscopy of HCV infected cells. In correlation to our earlier observations the electron microscopy data clearly suggests that HCV infection induces the perinuclear accumulation of damaged mitochondria followed by subsequent formation of mitophagosomes and mitophagolysosomes in HCV-infected cells (Figures 1B, 4D, 4E, 5B, and S1, S11). It should be noted that in HCV-infected cells, we observed a few mitophagolysosome at a given time point, because of the rapid turnover rate of the mitophagic process. Further, unlike any chemical treatment, extensive autophagic puncta were not seen in HCV-infected cell, suggesting that HCV tightly regulates the autophagic turnover. This observation is consistent with previous reports on HCV-associated autophagy [7]–[17]. Most importantly, in agreement with previous reports on bulk autophagy, specific inhibition of mitophagy was detrimental to viral replication (Figure 6). Parkin and PINK1 silencing affected viral RNA synthesis, thus implicating a functional role of mitophagy during HCV RNA replication process. HCV RNA synthesis is believed to occur on ER-derived membranous web like structures [3], but a detailed characterization of such structures is still lacking. Recent reports suggest that there is segregation of ATP pools at sites of HCV replication near cellular perinuclear region [59] and that autophagosomal membrane may serve as platforms for HCV replication [19]. In our study, HCV-infected cells displayed peculiar Parkin-dependent clustering of mitochondria near the perinuclear region as shown by confocal and electron microscopy. We surmise if such Parkin-dependent mitochondrial clustering serves to segregate ATP pools at HCV replication sties.

Previous studies have shown that HCV infection triggers ROS generation, which leads to loss of mitochondrial transmembrane potential (ΔΨ_m_) and decline in mitochondrial complex I activity [23], [24], [56]. A decrease in mitochondrial complex I activity in HCV infection has been shown to be a direct consequence of ROS generation and oxidation of mitochondrial glutathione pools and glutathionylation of mitochondrial complex I subunits [23]. Our results suggest that HCV induced Parkin-mediated mitophagy also causes the reduction of mitochondrial complex I activity in HCV infection. Knockdown of Parkin in HCV-infected cells restored the levels of mitochondrial complex I activity to the levels observed in uninfected cells (Figure 7). This return to normalcy may also be attributed to reduction of viral replication and protein synthesis in Parkin knockdown cells.

Chronic hepatitis B and C is associated with high mitochondrial ROS levels, ER ballooning and mitochondrial swelling [56], [60]. This observation lends support to our studies described here on mitophagy which marks damaged mitochondria for degradation. A recent report demonstrates a decrease in the number of mitochondria in the HCV-infected cells [8], [59]. Similarly, in our study, a decrease in mitochondria number in HCV infected cells was observed that was restored by treatment with both 3-MA and BafA1 (Figure 5E).

Autophagy and/or mitophagy appear to play an essential role during HCV infectious process. The process both removes damaged organelles and also promotes the survival and maintenance of persistently infected hepatocytes. Thus, these studies on selective mitophagy provide unique insight into the HCV associated liver disease pathogenesis and offer new avenues for the design of antiviral strategies.

## Materials and Methods

### Cell culture and virus

Human hepatoma cell lines Huh7 and Huh7.5.1 used in this study were grown in high-glucose DMEM (Gibco) supplemented with 10% fetal bovine serum (Hyclone), 1% MEM non-essential amino acids (Gibco), 100 units/ml penicillin (Gibco), and 100 µg/ml streptomycin (Gibco). Cell culture-derived HCV Jc1 genotype 2a (HCVcc) used in this study was propagated and prepared, as described previously [35]. HCVcc infection in this study was carried out at multiplicity of infection (MOI) of 1. Stable HCV replicon cells harboring full-length FLR-JFH1 (genotype 2a) and two subgenomic SGR-JFH1 (genotype 2a) and BM4–5 Feo (genotype 1b), respectively, were maintained in the presence of 0.4 mg/ml G418 (Invitrogen).

### Immunofluorescence

The cells were grown on glass cover slips, fixed in 4% paraformaldehyde, washed, and then permeabilized with 0.25% TritonX-100. MitoTracker CMXRos Red (Invitrogen) and LysoTracker (Invitrogen) were used to stain mitochondria and lysosomes in live cells before fixation. The cells were stained with the indicated antibodies. Wherever indicated, nuclei are stained with DAPI (Invitrogen). Images were visualized under a 100× oil objective using an Olympus FluoView 1000 confocal microscope. Quantification of images was conducted with ImageJ or MBF ImageJ softwares.

### DNA constructs

The pEGFP-LC3 plasmid DNA used in this study was a kind gift from Dr. Tamotsu Yoshimori (National Institute of Genetics, Japan). The pRK5-HA-Parkin plasmid DNA (plasmid ID: 17613) was purchased from Addgene.

### Reagents and antibodies

Chemical reagents used in this study were Bafilomycin A1 (Enzo Life Sciences), cyanide m-cholorophenyl hydrazone and 3-Methyladenine (Sigma). Primary antibodies used in this study include the following: rabbit monoclonal anti-LC3B (Cell Signaling); rabbit polyclonal anti-Parkin (Abcam); rabbit polyclonal anti-PINK1 (Abcam); rabbit polyclonal anti-VDAC1 (Cell Signaling); rabbit polyclonal anti-Calreticulin (Cell Signaling); rabbit polyclonal anti-GAPDH (SantaCruz); goat polyclonal anti-β-actin (SantaCruz); mouse monoclonal anti-TOM20 (BD); goat polyclonal anti-TOM20 (SantaCruz); rabbit polyclonal anti-TOM20 (Abcam); mouse monoclonal anti-Mitofusin2 (Mfn2) (Abcam); mouse monoclonal anti-SQSTM1/P62 (Abcam); mouse monoclonal anti-Ubiquitin (Cell Signaling); rabbit polyclonal anti-Ubiquitin (SantaCruz); mouse monoclonal anti-LAMP2 (Abcam); rabbit monoclonal anti-LAMP1 (Abcam); mouse monoclonal anti-HA (Roche); normal rabbit IgG (Cell Signaling); normal mouse IgG (SantaCruz); mouse monoclonal anti-human total OxPhos complex (Invitrogen); mouse monoclonal anti-HCV core (Thermo Scientific); human monoclonal anti-HCV E2 [61]; mouse monoclonal anti-HCV NS5A (clone 9E10). The secondary antibodies used for immunofluorescence were Alexa Fluor 350, 488, 594, or 647 donkey anti-mouse, rabbit, or goat IgG (Molecular Probe), Alexa Fluor 555 goat anti-human IgG (Molecular Probe), and Alexa Fluor 568 goat anti-mouse IgG (Molecular Probe). The secondary antibodies used for immunoblot analysis were HRP-conjugated anti-mouse IgG (Cell Signaling), HRP-conjugated anti-rabbit IgG (Cell Signaling), and HRP-conjugated anti-goat IgG (Jackson Laboratories).

### Transfection of siRNA

siGENOME SMARTpool small interfering RNA (siRNA) for Parkin (NM_004562), PINK1 (NM_032409), ATG5 (NM_004849), and non-targeting #1 control (NT) were used in this study (Dharmacon). Huh7 cells were transfected with siRNAs (50 nM) using DharmaFECT 4 transfection reagent according to the manufacturer's instructions (Dharmacon), prior to HCVcc infection.

### Establishment of Huh7 cells stably expressing Parkin-shRNA

To establish stable human hepatoma cell line expressing Parkin-specific shRNA (P-KD), Huh7 cells were transfected with shRNA construct (pLKO.1-puro/Parkin, Sigma) encoding siRNA targeting Parkin using TransIT-LT1 transfection reagent (Mirus, Madison, WI) according to the manufacturer's instructions and subsequently, selected in the presence of 5 µg/ml of puromycin for 3 weeks. Parkin shRNA sequence is as follows: TRCN0000000281; CCGGCGTGAACATAACTGAGGGCATCTCGAGATGCCCTCAGTTATGTTCACGTTTTT. Two stable M-KD and NT-KD cell lines expressing empty vector (pLKO.1-puro, Sigma) and nontargeting shRNA (pLKO.1-puro/non-targeting, Sigma), respectively, were also established as a negative controls. All cells were maintained in the presence of 2.5 µg/ml of puromycin. The knockdown level of Parkin gene was analyzed by immunoblotting with a specific antibody against Parkin.

### Immunoprecipitation and Western blot analysis

For Western blot analysis, cells were resuspended in RIPA buffer (20 mM Tris-HCl [pH 7.5], 150 mM NaCl, 50 mM NaF, 1 mM Na_3_VO_4_, 0.1% SDS, and 0.5% TritonX-100) supplemented with a Halt protease inhibitor cocktail (Thermo Scientific). The whole cell lysates (WCL) were subjected to SDS-PAGE, transferred to nitrocellulose membrane (Thermo Scientific), and Western blot analyzed with antibodies against the indicated proteins.

For immunoprecipitation of the ubiquitinated Parkin in the pure mitochondria fraction, 100 µg of the purified cytosolic and mitochondrial fractions were resuspended in RIPA buffer without SDS. The resuspended mixtures were immunoprecipitated with anti-Parkin antibody and protein-G Sepharose (GE Healthcare) followed by Western blotting with anti-ubiquitin antibody.

For immunoprecipitation of the ubiquitinated Parkin, Mfn2, and VDAC1 in WCL, Huh7 cells infected with HCVcc were suspended in 0.1 ml of RIPA buffer. The suspended cells were incubated for 20 min on ice and clarified by centrifugation at 15,000×g at 4°C for 20 min. The supernatant was mixed with 1.9 ml of RIPA buffer without SDS and immunoprecipitated with anti-ubiquitin, Mfn2, and VDAC1 antibodies, respectively, and protein-G Sepharose followed by Western blotting with anti-Parkin and ubiquitin antibodies, respectively.

### Real-time RT-qPCR

To analyze the expression levels of Parkin, PINK1, and ATG5 genes, total cellular RNA was extracted from cells using RNAeasy Mini kit (Qiagen) and subsequently, complementary DNAs (cDNAs) was synthesized by using SuperScript III First-Strand Synthesis System with oligo(dT)_20_ primer (Invitrogen) according to the manufacturer's instructions, respectively. DyNAmo HS SYBR Green qPCR kit (Finnzymes) was used to quantify the cellular RNA levels. The following primer sets were used for RT-PCR: ATF4 forward, 5′-AGTCCCTCCAACAACAGCAA; ATF4 reverse, 5′-GAAGGTCATCTGGCATGGTT; Parkin forward, 5′-TACGTGCACAGACGTCAGGAG; Parkin reverse, 5′-GACAGCCAGCCACACAAGGC; PINK1 forward, 5′-GGGGAGTATGGAGCAGTCAC; PINK1 reverse, 5′-CATCAGGGTAGTCGACCAGG; ATG5 forward, 5′-GCCATCAATCGGAAACTCAT; ATG5 reverse, 5′-ACTGTCCATCTGCAGCCAC; GAPDH forward, 5′-GCCATCAATGACCCCTTCATT; and GAPDH reverse, 5′-TTGACGGTGCCATGGAATTT. HCV RNA levels were quantified by qRT-PCR, as described previously [35].

To analyze the expression levels of mitochondrial DNA, total cellular DNA was extracted from the cells using AllPrep DNA kit (Qiagen) and subsequently quantified by qPCR using DyNAmo HS SYBR Green qPCR kit according to the manufacturer's instructions. The following primer sets were used for qPCR: ND-2 forward, 5′-TAGCCCCCTTTCACTTCTGA; ND-2 reverse, 5′-GCGTAGCTGGGTTTGGTTTA; COX-2 forward, 5′-GGCCACCAATGGTACTGAAC; COX-2 reverse, 5′-CGGGAATTGCATCTGTTTTT. Real-time qPCR was conducted by using an ABI PRISM 7000 Sequence Detection System (Applied Biosystems).

### Subcellular fractionation

To isolate pure mitochondrial fraction from HCV-infected cells, Huh7 cells were infected with HCVcc. At 5 days post-infection, cells were homogenized and then pure cytosolic and mitochondrial fractions were isolated by Percoll gradient fractionation, as described previously [62]. Equivalent amounts of protein from each fraction were analyzed by Western blotting with the indicated antibodies.

### Measurement of mitochondrial complex I enzyme activity

The activity of mitochondrial oxidative phosphorylation respiratory chain complex I (NADH dehydrogenase) in HCV-infected cells was measured by using mitochondrial complex I activity assay kit according to the manufacturer's instructions (Novagen). Briefly, Huh7, NT-KD, and P-KD cells were infected with HCVcc for 3 days. Huh7 cells were treated with 3-MA (10 mM) and BafA1 (100 nM) for 12 h before harvest. 500 µg of the detergent-soluble WCL were used for this assay.

### Cell viability assay

To assess cytotoxic effects of Parkin silencing during HCV infection, NT-KD and P-KD cells infected with HCVcc for 3 days were incubated with 10% resazurin solution (TOX-8, Sigma) for 4 hours at 37°C and then cell viability was measured according to the manufacturer's instructions.

### Electron microscopy and immunoelectron microscopy

Briefly, Huh7 and HCV-infected cells grown in 10 cm dishes were washed and fixed with fixative containing 2% glutaraldehyde in 0.1 M sodium cacodylate buffer [pH 7.4]. Cell pellets were embedded in 2% agarose, post-fixed with 1% osmium tetroxide, and dehydrated with an acetone series. Samples were infiltrated, embedded in Durcupan and polymerized at 60°C for 48 h. Ultrathin sections were prepared and examined using a JEOL 1200 EX II transmission electron microscope at 80 kV. HCV infected cells were stained with indicated antibodies and treated with secondary antibodies conjugated with indicated immunogold particles for immunoelectron microscopy.

### Human liver biopsy specimens

The frozen human liver biopsy specimens (*n* = 7) were a kind gift from Dr. Tarek Hassanein, UCSD hepatology clinic. The liver biopsy specimens used in this study include the following: anti-HCV negative normal patient, *n* = 1; anti-HCV negative patient with hepatocarcinoma, *n* = 1; anti-HCV positive patients with chronic HCV including 3 females and 2 male, *n* = 5.

### Statistical analysis

Statistical analyses using unpaired Student's t-test were performed by using Sigma Plot software (Systat Software Inc., San Jose, CA, USA).

## Supporting Information

Figure S1 **HCV infection induces mitochondrial damage and perinuclear clustering.** (**A**, **B**) Huh7 cells were infected with HCVcc. At 3 days post-infection, cells were fixed and examined by electron microscopy. (**A**) Ultrastructure of HCV-infected cells showing the perinuclear clustering of damaged mitochondria. In the zoomed image, abnormality of ultrastructural mitochondria with loss of mitochondrial cristae in HCV-infected cells is shown. Organelle marker: N, nucleus; white arrow, mitochondria. Scale bar = 1 µM. (**B**) Confocal images showing Huh7 cells infected with HCVcc for electron microscopy of the panel (**A**). Cells were immunostained with anti-HCV core antibody (red). Nuclei were stained with DAPI (blue). (**C**, **D**) Huh7 cells infected with HCVcc were processed immuno-EM with anti-TOM20 antibody. (**C**) Ultrastructure of HCV-infected cells showing the perinuclear clustering of damaged mitochondria. In the zoomed image, the gold particles indicate TOM20 (white arrow, 12 nm) in the damaged mitochondria with the loss of mitochondrial cristae in HCV-infected cells. Organelle marker: N, nucleus; M, mitochondria. Scale bar = 200 nM. (**D**) Confocal images showing Huh7 cells infected with HCVcc for immuno-EM of the panel (**C**). Cells were immunostained with anti-HCV core antibody (red). Nuclei were stained with DAPI (blue).
(PDF)

Figure S2 **CCCP induces the mitochondrial translocation of Parkin in human hepatoma Huh7 cells.** Confocal microscopy showing Parkin aggregates on the mitochondrial perinuclear clusters of CCCP-treated cells. Huh7 cells were treated with CCCP (10 µM). At 12 h post-treatment, cells prestained with MitoTracker (Mito, red) were immunostained with anti-Parkin (green) antibody. Nuclei were stained with DAPI (blue). In the zoomed images, the yellow color indicates endogenous Parkin aggregates on the mitochondria.
(PDF)

Figure S3 **HCV induces the mitochondrial translocation of Parkin in HCV full-length or subgenomic replicon-bearing cells.** (**A**–**D**) Representative confocal images showing endogenous Parkin translocation to the mitochondrial perinuclear clusters in cells stably expressing HCV replicons. Stable cells harboring HCV full-length replicon FLR-JFH1 (genotype 2a), subgenomic replicon SGR-JFH1 (genotype 2a), and subgenomic replicon BM4–5 Feo (genotype 1b), respectively, and human hepatoma Huh7.5.1 cells were immunostained with anti-Parkin antibody. MitoTracker (Mito) was used for staining live mitochondria before fixation. The expression of HCV proteins (light gray) is verified by immunostaining with anti-HCV core (**A**) or NS5A antibody (**B**, **C**, and **D**). Nuclei were stained with DAPI (blue). In the zoomed images, yellow color indicates the colocalization of Parkin (green) with mitochondria (red). (**E**) ImageJ quantification of Parkin associated with mitochondria is described (mean ± SEM; *n*≥10 cells, *p<0.001). P values were calculated by using an unpaired Student's t-test.
(PDF)

Figure S4 **HCV-induced Parkin-mediated ubiquitination of Mfn2.** (**A**) Representative confocal images showing the ubiquitination of Mfn2 in HCV-infected cells. At 2 days post-infection, Huh7 cells infected with HCVcc were immunostained with anti-Mfn2 (green), Ub (red), and HCV E2 (light gray) antibodies. Nuclei are demarcated with white dot circles. In the zoomed images, the arrows indicate the ubiquitination of endogenous Mfn2 (yellow spots). (**B**) ImageJ quantitative analysis of the ubiquitination of endogenous Mfn2 (mean ± SEM; n≥10 cells; *p<0.05). P values were calculated by using an unpaired Student's t-test.
(PDF)

Figure S5 **HCV-induced Parkin-mediated ubiquitination of VDAC1.** (**A**) Representative confocal images showing the ubiquitination of VDAC1 in HCV-infected cells. At 2 days post-infection, Huh7 cells infected with HCVcc were immunostained with anti-VDAC1 (green), Ub (red), and HCV E2 (light gray) antibodies. Nuclei are demarcated with white dot circles. In the zoomed images, the arrows indicate the ubiquitination of endogenous VDAC1 (yellow spots). (**B**) ImageJ quantitative analysis of the ubiquitination of endogenous VDAC1 (mean ± SEM; n≥10 cells; *p<0.01). P values were calculated by using an unpaired Student's t-test.
(PDF)

Figure S6 **HCV infection induces the interaction between Parkin and p62 associated with mitochondria.** (**A**) Representative confocal images showing the colocalization of Parkin and p62 on mitochondria in HCV-infected cells. At 2 days post-infection, HCV-infected cells prestained with MitoTracker (Mito) were immunostained with anti-p62 (green), Parkin (red), and HCV E2 (light gray) antibodies. Nuclei are demarcated with white dot circles. In the zoomed images, the arrows indicate the colocalization of endogenous p62 and Parkin on mitochondria (white spots). (**B**) ImageJ quantitative analysis of the merge of endogenous p62 and Parkin associated with mitochondria (mean ± SEM; n≥10 cells; *p<0.05). P values were calculated by using an unpaired Student's t-test.
(PDF)

Figure S7 **HCV infection enhances the ubiquitination of the autophagy-associated factor, p62.** (**A**) Representative confocal images showing the ubiquitination of p62 on mitochondria in HCV-infected cells. At 2 days post-infection, HCV-infected cells prestained with MitoTracker (Mito) were immunostained with anti-p62 (green), Ub (blue), and HCV E2 (light gray) antibodies. Nuclei are demarcated with white dot circles. In the zoomed images, the arrows indicate the ubiquitination of endogenous p62 on mitochondria (white spots). (**B**) ImageJ quantitative analysis of the ubiquitination of endogenous p62 on mitochondria (mean ± SEM; n≥10 cells; *p<0.05). P values were calculated by using an unpaired Student's t-test.
(PDF)

Figure S8 **Knockdown of Parkin attenuates HCV-induced mitophagy.** (**A**) Confocal microscopy showing the formation of mitophagosome in the cells expressing non-targeting shRNA (NT-KD) or Parkin-specific shRNA (P-KD) infected with HCVcc. NT-KD and P-KD cells transiently expressing GFP-LC3 protein (green) were infected with HCVcc. At 2 days post-infection, cells prestained with Mitotracker (Mito, red) were immunostained with anti-Parkin (orange) and HCV core (cyan) antibodies. Nuclei are demarcated with white dot circles. Infected (+) and uninfected (−) cells are marked. (**B**) Quantification of the number of GFP-LC3 puncta colocalized with mitochondria in the panel (**A**) (mean ± SEM; n≥10 cells; *p<0.01). P values were calculated by using an unpaired Student's t-test.
(PDF)

Figure S9 **BafA1 treatment promotes mitophagosome accumulation in Huh7 cells.** (**A**) Confocal microscopy showing the accumulation of mitophagosome in the presence of BafA1. Huh7 cells transiently expressing GFP-LC3 protein (green) were treated with BafA1 for 12 h before fixation. Cells prestained with Mitotracker (Mito, red) were immunostained with anti-Parkin (cyan) and HCV core (light gray) antibodies. Nuclei are demarcated with white dot circles. (**B**) Quantification of the number of GFP-LC3 puncta colocalized with mitochondria in the panel (**A**) (mean ± SEM; n≥10 cells; *p<0.05). P values were calculated by using an unpaired Student's t-test.
(PDF)

Figure S10 **HCV induces the formation of Parkin-containing mitophagolysosome.** Confocal microscopy showing the formation of Parkin-containing mitophagolysosome in HCV-infected cells. Huh7 cells transiently expressing GFP-LC3 protein (green) were infected with HCVcc in the absence or presence of 3-MA (10 mM) and BafA1 (100 nM), respectively, for 12 h before fixation. At 3 days post-infection, cells were immunostained with antibodies against Parkin (blue), LAMP2 (red), and HCV E2 (cyan). Nuclei are demarcated with white dot circles. In the zoomed images, the arrows indicate the colocalization of GFP-LC3 puncta, lysosome, and Parkin in HCV-infected cells (white spots).
(PDF)

Figure S11 **Immunoelectron microscopy of HCV infected cells.** (**A**–**C**) Huh7 cells were infected with HCVcc. At 3 days post-infection, cells were fixed, processed for immuno-EM with the indicated antibodies, and examined by electron microscopy. (**A**) Ultrastructure of HCV-infected cells showing the mitochondrial translocation of Parkin. In the zoomed image, the gold particles indicate TOM20 (black arrow, 18 nm), HCV E2 (red arrow, 12 nm), and Parkin (blue arrow, 6 nm) in the damaged mitochondria with loss of mitochondrial cristae in HCV-infected cells. (**B**) Ultrastructure of HCV-infected cells showing the formation of mitophagosome. In the zoomed image, the gold particles indicate GFP-LC3 (black arrow, 18 nm), HCV E2 (red arrow, 12 nm), and TOM20 (blue arrow, 6 nm) in the damaged mitochondria of HCV-infected cells. (**C**) Ultrastructure of HCV-infected cells showing the formation of mitophagolysosome containing Parkin. In the zoomed image, the gold particles indicate LAMP1 (black arrow, 18 nm), HCV E2 (red arrow, 12 nm), and Parkin (blue arrow, 6 nm) in the fusion of damaged mitochondria-containing vesicles with the lysosome in HCV-infected cells. The mitophagolysosome formation is demarcated with white dot circle. Organelle marker: N, nucleus; M, mitochondria; L, lysosome. Scale bar = 200 nM.
(PDF)

Figure S12 **Viability of Parkin knockdown cells after infection with HCVcc.** NT-KD and P-KD cells were infected with HCVcc. At 3 days post-infection, cell viability was measured as described in Materials and Methods. Cell viability is expressed as the percentage of the viable cells in the infected cells relative to the uninfected controls (mean ± SD; n = 3; *p<0.05). P values were calculated by using an unpaired Student's t-test.
(PDF)
