# Supplementary figures and images for: Hepatitis C Virus Induces the Mitochondrial Translocation of Parkin and Subsequent Mitophagy
Source: PLoS Pathog. 2013 Mar 28;9(3):e1003285. doi: 10.1371/journal.ppat.1003285 (PMC3610669; doi:10.1371/journal.ppat.1003285)

**Figure S1**

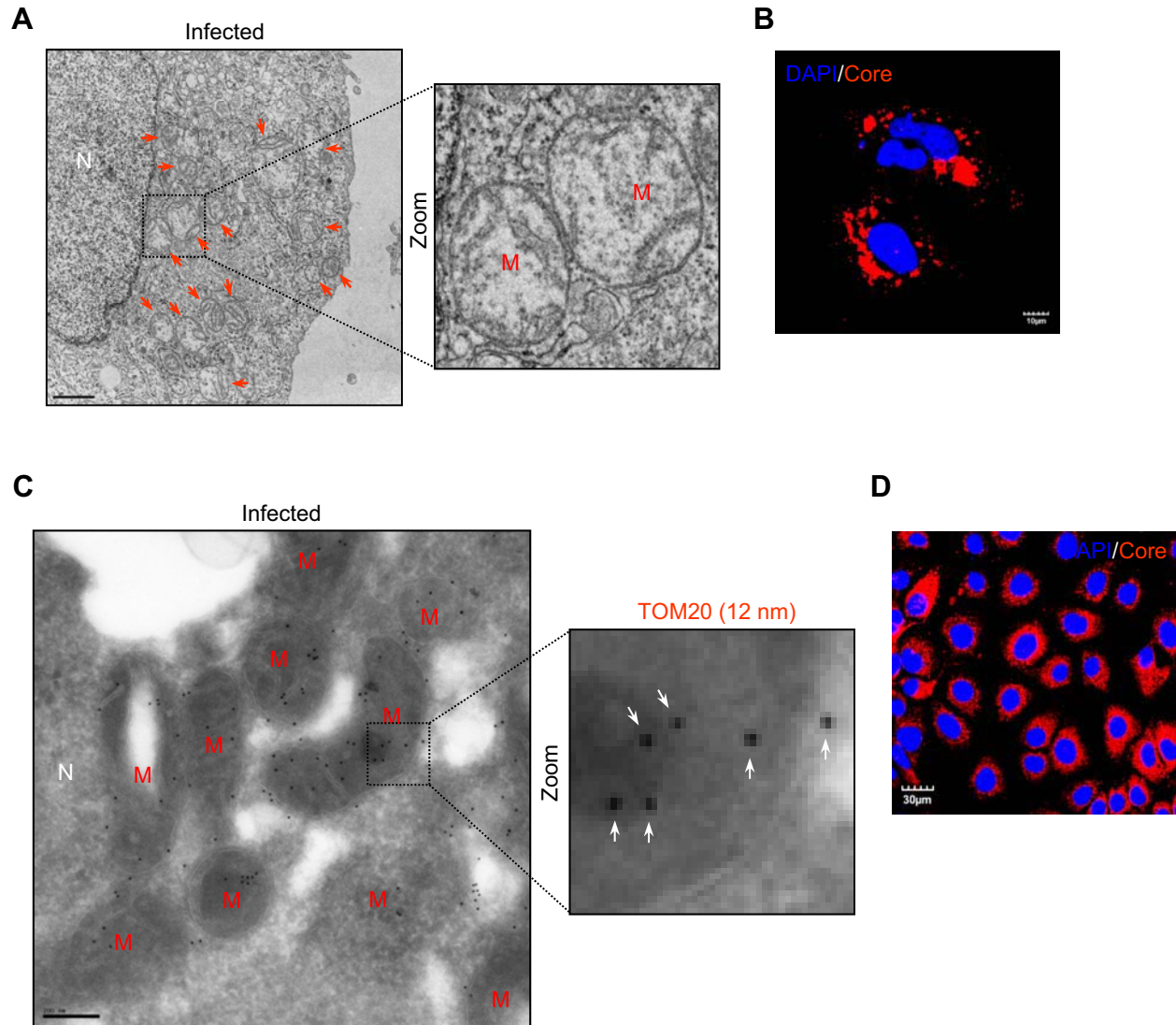

Supplement: Figure S1 — HCV infection induces mitochondrial damage and perinuclear clustering. (A, B) Huh7 cells were infected with HCVcc. At 3 days post-infection, cells were fixed and examined by electron microscopy. (A) Ultrastructure of HCV-infected cells showing the perinuclear clustering of damaged mitochondria. In the zoomed image, abnormality of ultrastructural mitochondria with loss of mitochondrial cristae in HCV-infected cells is shown. Organelle marker: N, nucleus; white arrow, mitochondria. Scale bar = 1 µM. (B) Confocal images showing Huh7 cells infected with HCVcc for electron microscopy of the panel (A). Cells were immunostained with anti-HCV core antibody (red). Nuclei were stained with DAPI (blue). (C, D) Huh7 cells infected with HCVcc were processed immuno-EM with anti-TOM20 antibody. (C) Ultrastructure of HCV-infected cells showing the perinuclear clustering of damaged mitochondria. In the zoomed image, the gold particles indicate TOM20 (white arrow, 12 nm) in the damaged mitochondria with the loss of mitochondrial cristae in HCV-infected cells. Organelle marker: N, nucleus; M, mitochondria. Scale bar = 200 nM. (D) Confocal images showing Huh7 cells infected with HCVcc for immuno-EM of the panel (C). Cells were immunostained with anti-HCV core antibody (red). Nuclei were stained with DAPI (blue). (PDF) [file ppat.1003285.s001.pdf]

Figure S2

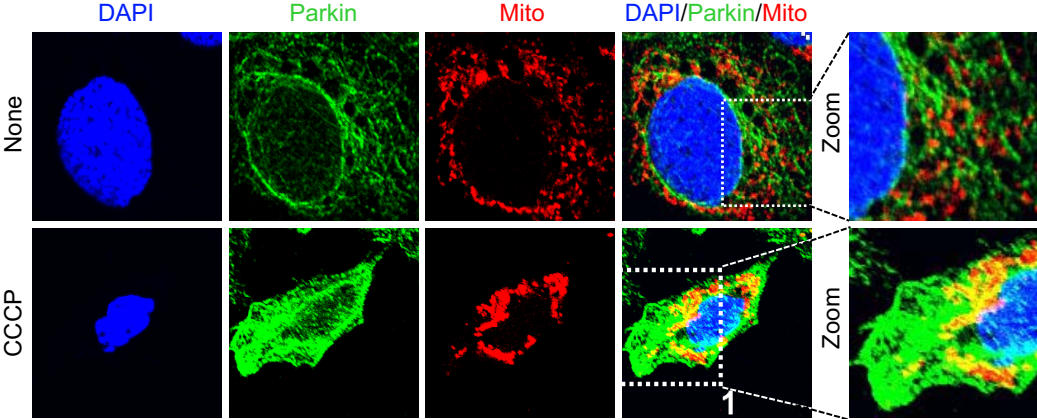

Supplement: Figure S2 — CCCP induces the mitochondrial translocation of Parkin in human hepatoma Huh7 cells. Confocal microscopy showing Parkin aggregates on the mitochondrial perinuclear clusters of CCCP-treated cells. Huh7 cells were treated with CCCP (10 µM). At 12 h post-treatment, cells prestained with MitoTracker (Mito, red) were immunostained with anti-Parkin (green) antibody. Nuclei were stained with DAPI (blue). In the zoomed images, the yellow color indicates endogenous Parkin aggregates on the mitochondria. (PDF) [file ppat.1003285.s002.pdf]

Figure S4

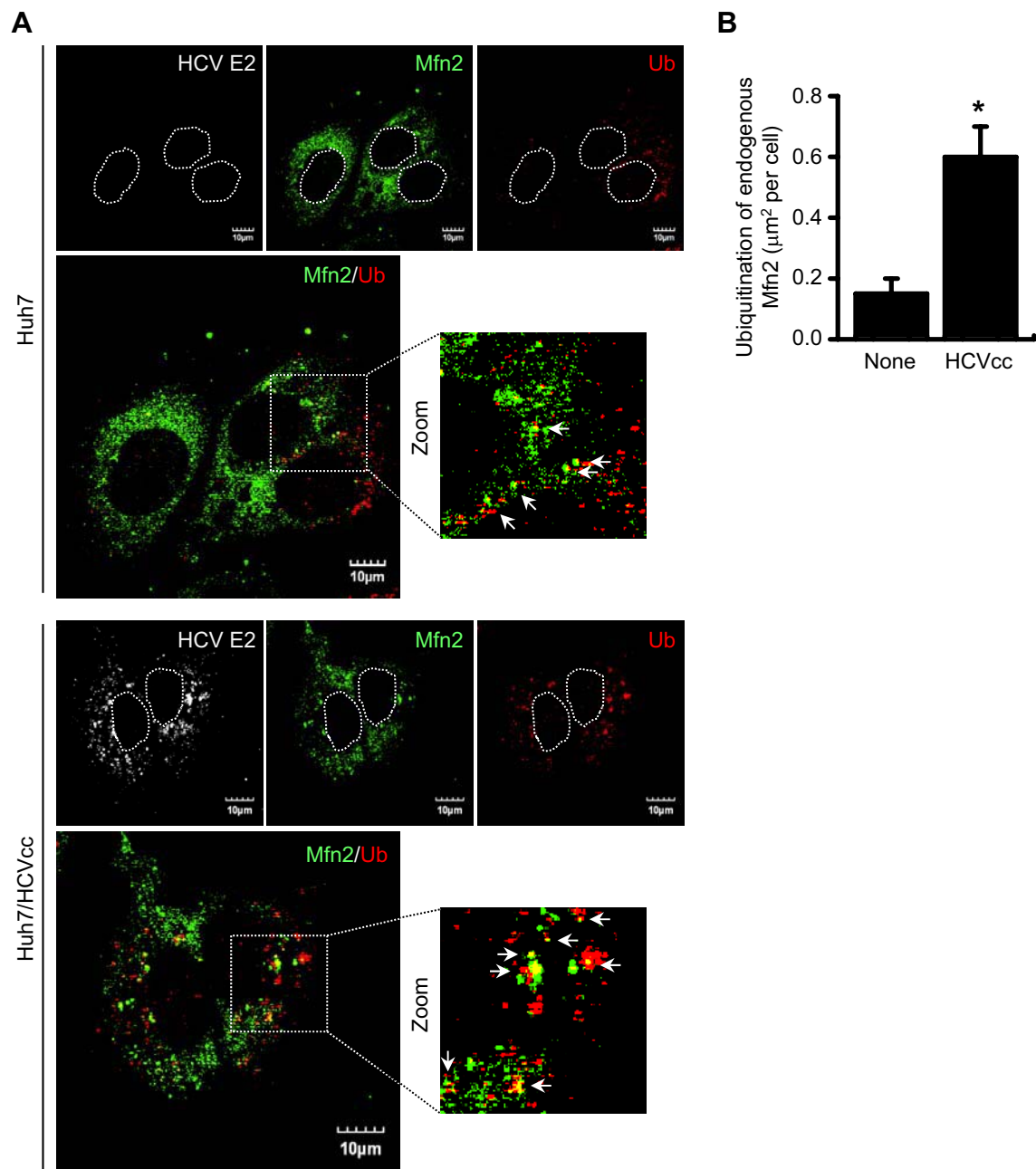

Supplement: Figure S4 — HCV-induced Parkin-mediated ubiquitination of Mfn2. (A) Representative confocal images showing the ubiquitination of Mfn2 in HCV-infected cells. At 2 days post-infection, Huh7 cells infected with HCVcc were immunostained with anti-Mfn2 (green), Ub (red), and HCV E2 (light gray) antibodies. Nuclei are demarcated with white dot circles. In the zoomed images, the arrows indicate the ubiquitination of endogenous Mfn2 (yellow spots). (B) ImageJ quantitative analysis of the ubiquitination of endogenous Mfn2 (mean ± SEM; n≥10 cells; *p<0.05). P values were calculated by using an unpaired Student's t-test. (PDF) [file ppat.1003285.s004.pdf]

**Figure S5**

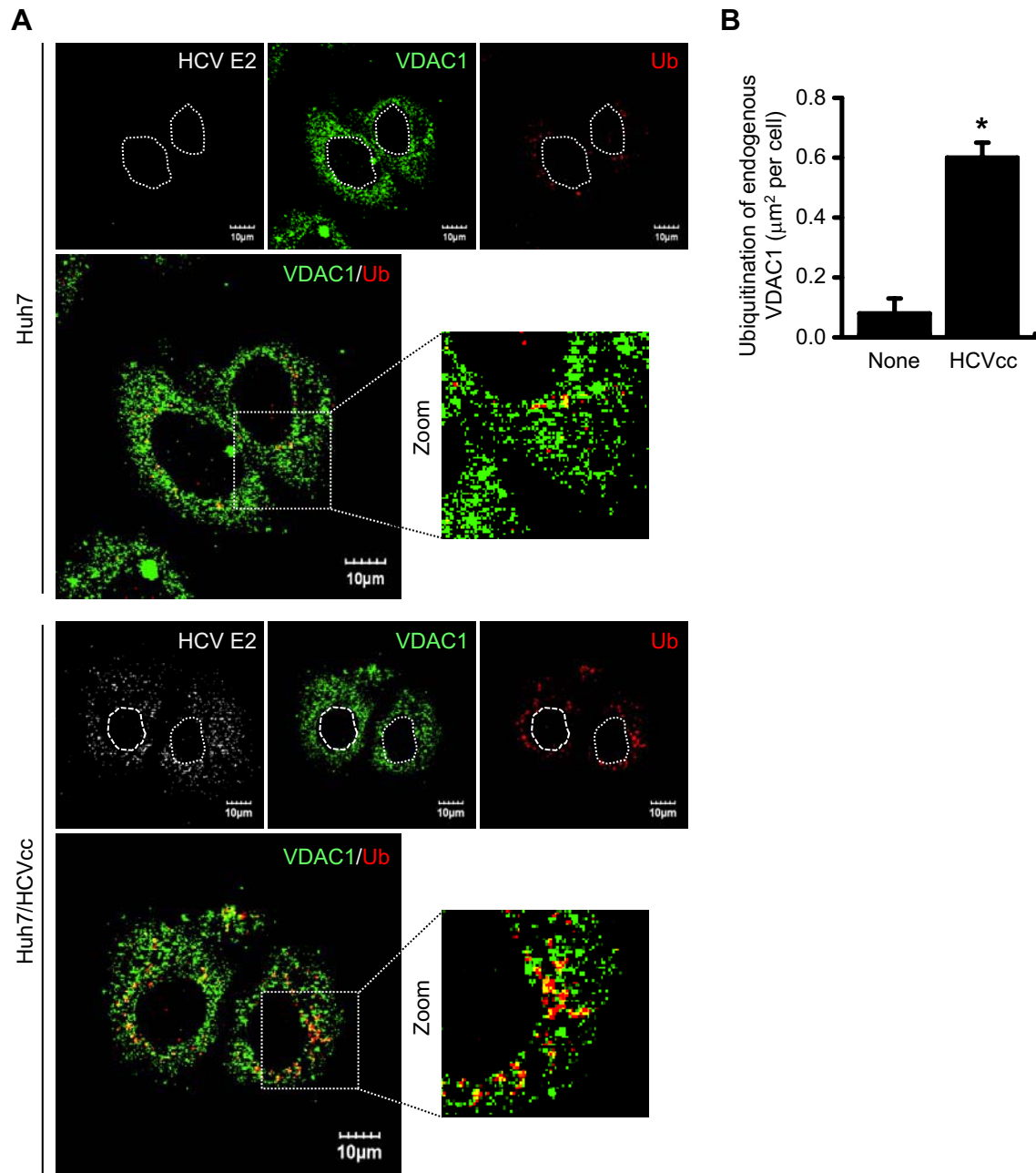

Supplement: Figure S5 — HCV-induced Parkin-mediated ubiquitination of VDAC1. (A) Representative confocal images showing the ubiquitination of VDAC1 in HCV-infected cells. At 2 days post-infection, Huh7 cells infected with HCVcc were immunostained with anti-VDAC1 (green), Ub (red), and HCV E2 (light gray) antibodies. Nuclei are demarcated with white dot circles. In the zoomed images, the arrows indicate the ubiquitination of endogenous VDAC1 (yellow spots). (B) ImageJ quantitative analysis of the ubiquitination of endogenous VDAC1 (mean ± SEM; n≥10 cells; *p<0.01). P values were calculated by using an unpaired Student's t-test. (PDF) [file ppat.1003285.s005.pdf]

Figure S6

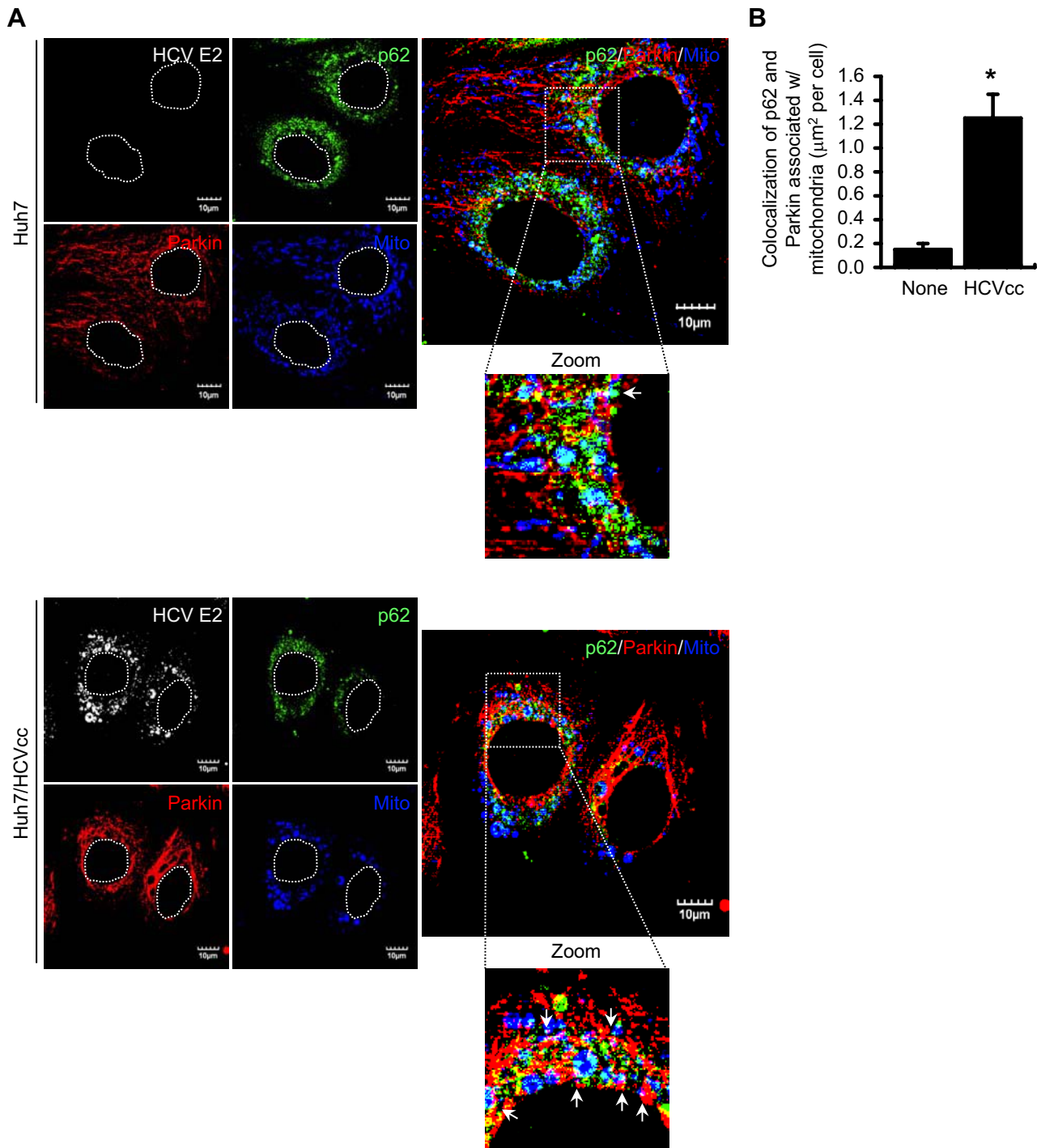

Supplement: Figure S6 — HCV infection induces the interaction between Parkin and p62 associated with mitochondria. (A) Representative confocal images showing the colocalization of Parkin and p62 on mitochondria in HCV-infected cells. At 2 days post-infection, HCV-infected cells prestained with MitoTracker (Mito) were immunostained with anti-p62 (green), Parkin (red), and HCV E2 (light gray) antibodies. Nuclei are demarcated with white dot circles. In the zoomed images, the arrows indicate the colocalization of endogenous p62 and Parkin on mitochondria (white spots). (B) ImageJ quantitative analysis of the merge of endogenous p62 and Parkin associated with mitochondria (mean ± SEM; n≥10 cells; *p<0.05). P values were calculated by using an unpaired Student's t-test. (PDF) [file ppat.1003285.s006.pdf]

Figure S7

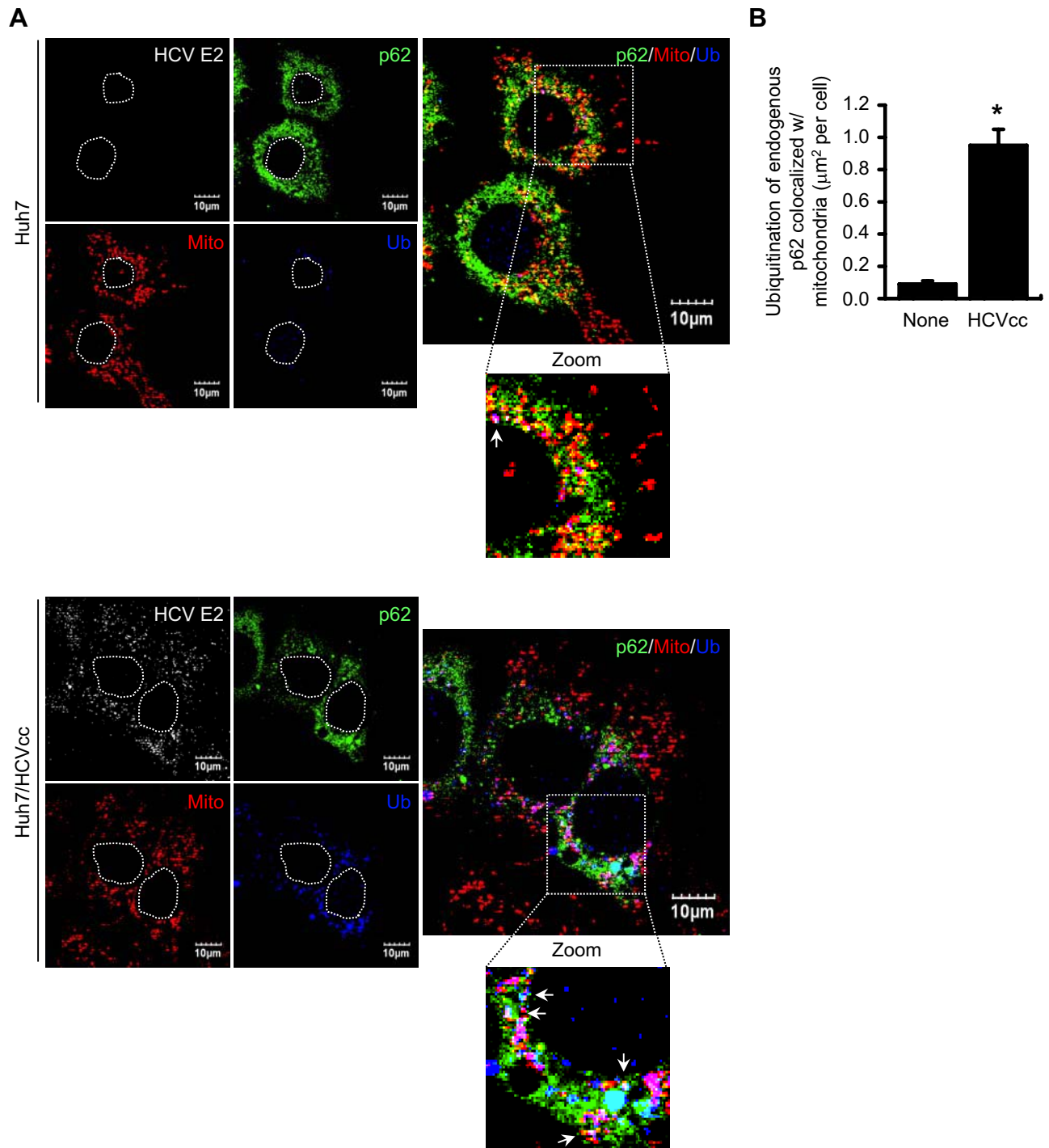

Supplement: Figure S7 — HCV infection enhances the ubiquitination of the autophagy-associated factor, p62. (A) Representative confocal images showing the ubiquitination of p62 on mitochondria in HCV-infected cells. At 2 days post-infection, HCV-infected cells prestained with MitoTracker (Mito) were immunostained with anti-p62 (green), Ub (blue), and HCV E2 (light gray) antibodies. Nuclei are demarcated with white dot circles. In the zoomed images, the arrows indicate the ubiquitination of endogenous p62 on mitochondria (white spots). (B) ImageJ quantitative analysis of the ubiquitination of endogenous p62 on mitochondria (mean ± SEM; n≥10 cells; *p<0.05). P values were calculated by using an unpaired Student's t-test. (PDF) [file ppat.1003285.s007.pdf]

**Figure S8**

**A**

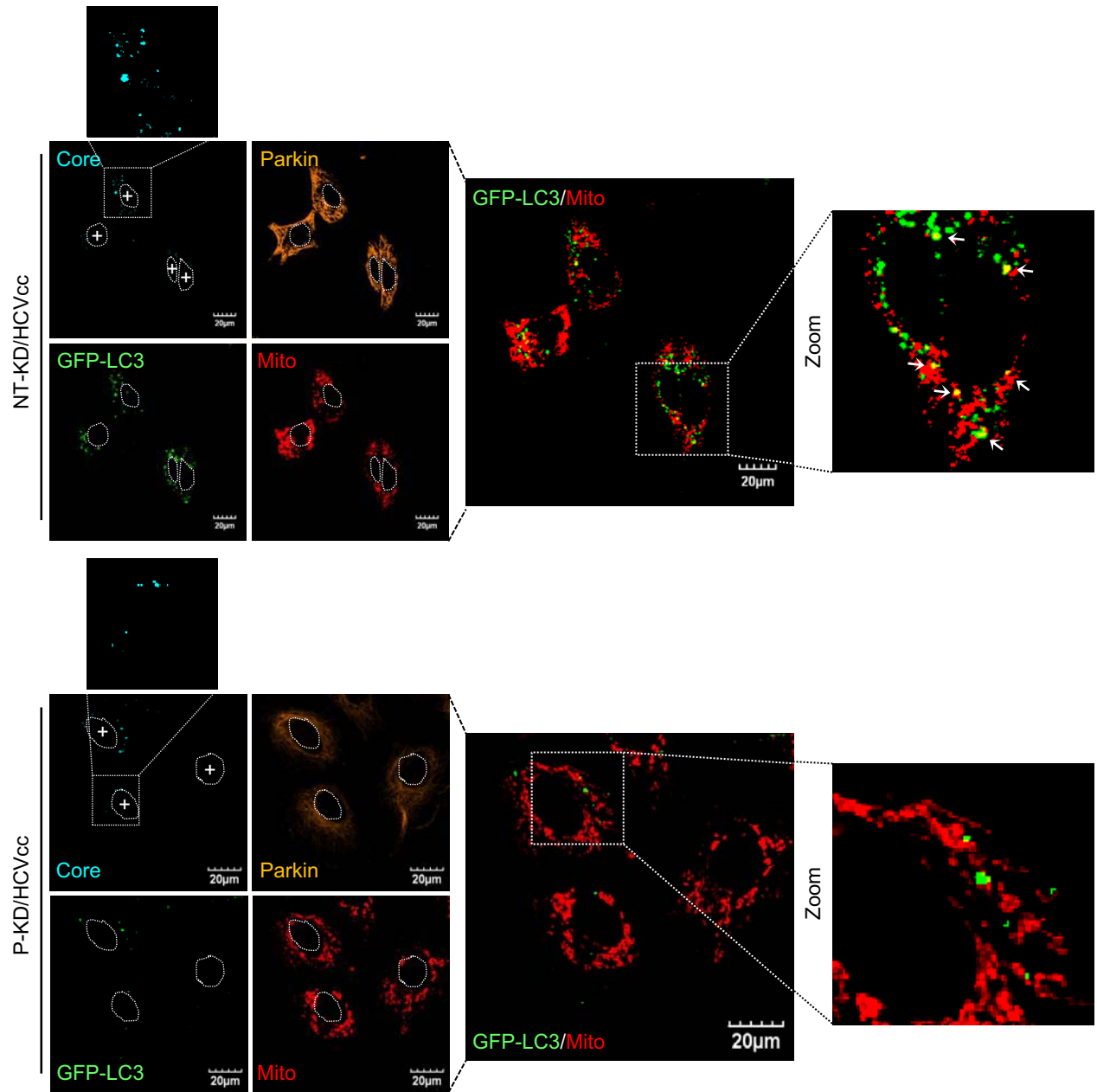

**B**

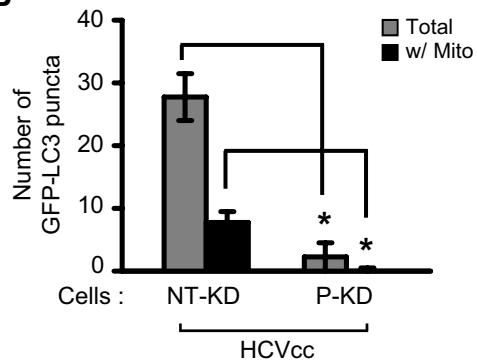

Supplement: Figure S8 — Knockdown of Parkin attenuates HCV-induced mitophagy. (A) Confocal microscopy showing the formation of mitophagosome in the cells expressing non-targeting shRNA (NT-KD) or Parkin-specific shRNA (P-KD) infected with HCVcc. NT-KD and P-KD cells transiently expressing GFP-LC3 protein (green) were infected with HCVcc. At 2 days post-infection, cells prestained with Mitotracker (Mito, red) were immunostained with anti-Parkin (orange) and HCV core (cyan) antibodies. Nuclei are demarcated with white dot circles. Infected (+) and uninfected (−) cells are marked. (B) Quantification of the number of GFP-LC3 puncta colocalized with mitochondria in the panel (A) (mean ± SEM; n≥10 cells; *p<0.01). P values were calculated by using an unpaired Student's t-test. (PDF) [file ppat.1003285.s008.pdf]

Figure S9

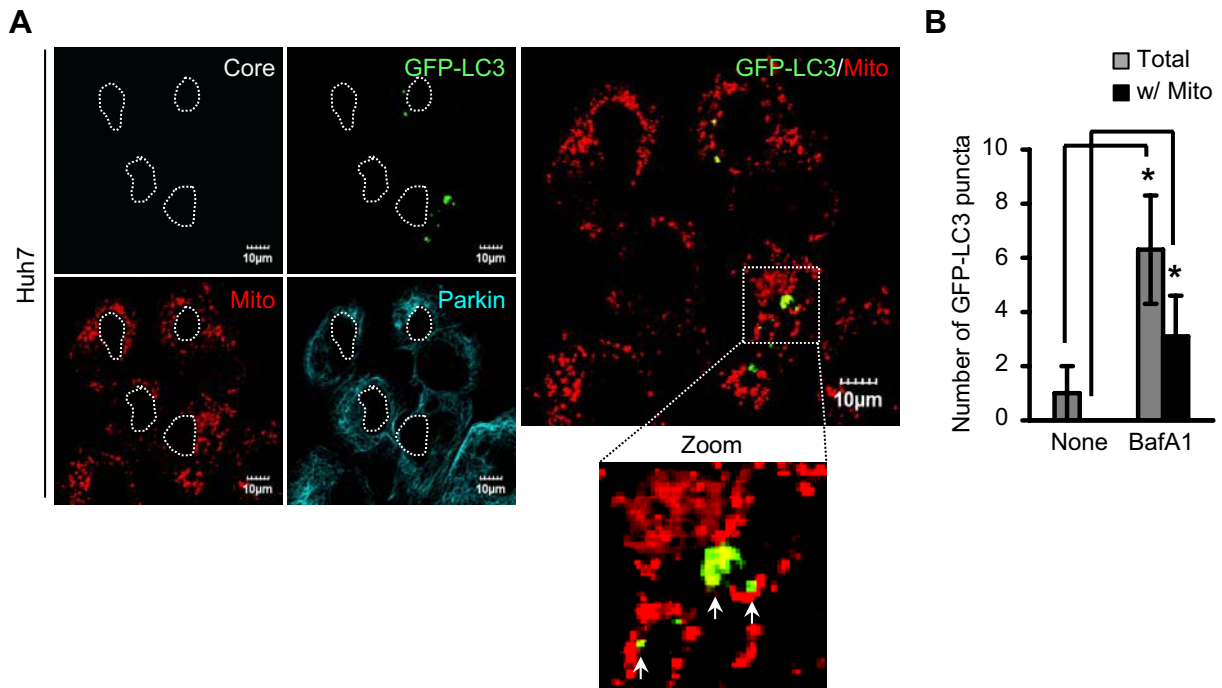

Supplement: Figure S9 — BafA1 treatment promotes mitophagosome accumulation in Huh7 cells. (A) Confocal microscopy showing the accumulation of mitophagosome in the presence of BafA1. Huh7 cells transiently expressing GFP-LC3 protein (green) were treated with BafA1 for 12 h before fixation. Cells prestained with Mitotracker (Mito, red) were immunostained with anti-Parkin (cyan) and HCV core (light gray) antibodies. Nuclei are demarcated with white dot circles. (B) Quantification of the number of GFP-LC3 puncta colocalized with mitochondria in the panel (A) (mean ± SEM; n≥10 cells; *p<0.05). P values were calculated by using an unpaired Student's t-test. (PDF) [file ppat.1003285.s009.pdf]

Figure S10

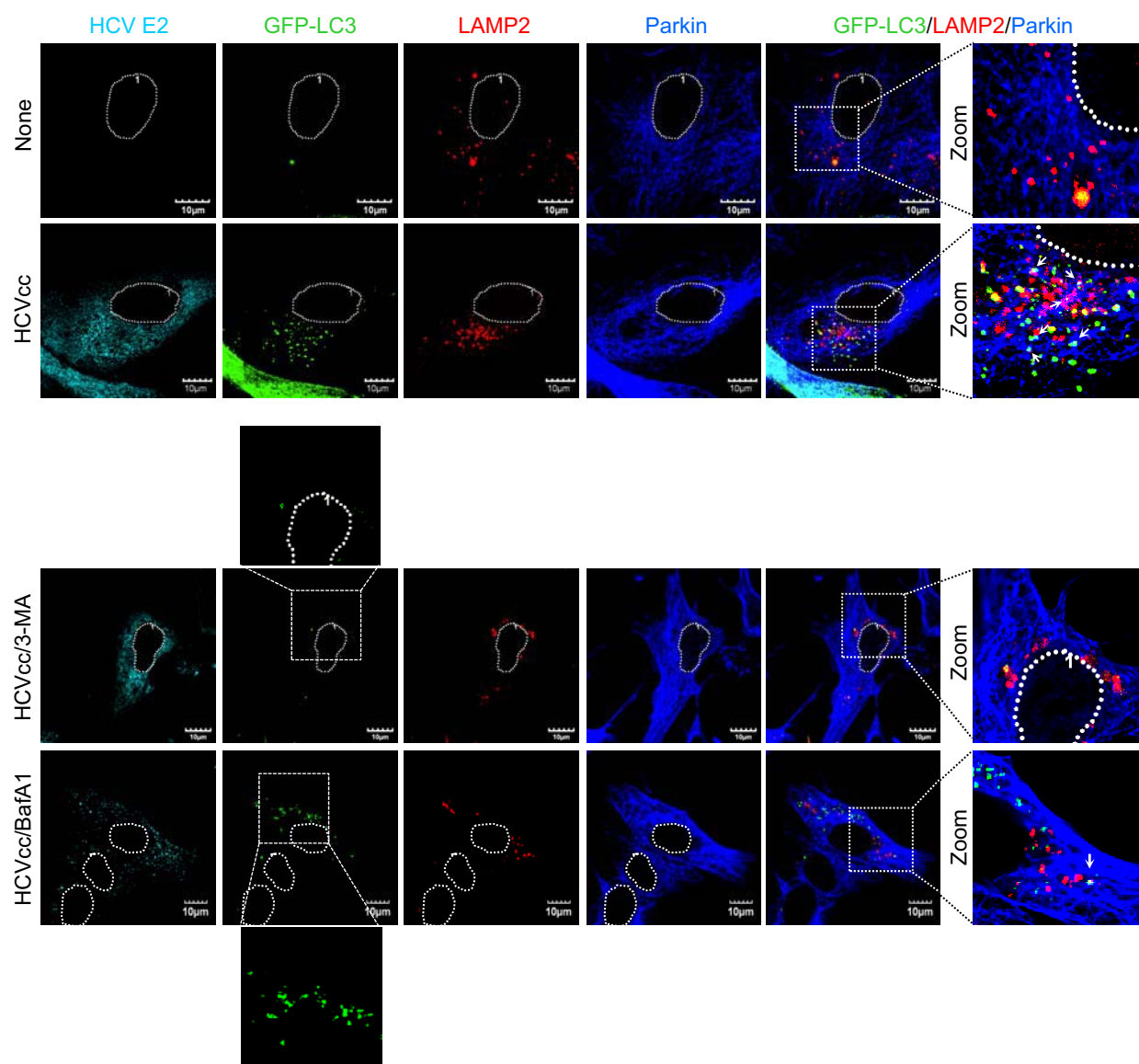

Supplement: Figure S10 — HCV induces the formation of Parkin-containing mitophagolysosome. Confocal microscopy showing the formation of Parkin-containing mitophagolysosome in HCV-infected cells. Huh7 cells transiently expressing GFP-LC3 protein (green) were infected with HCVcc in the absence or presence of 3-MA (10 mM) and BafA1 (100 nM), respectively, for 12 h before fixation. At 3 days post-infection, cells were immunostained with antibodies against Parkin (blue), LAMP2 (red), and HCV E2 (cyan). Nuclei are demarcated with white dot circles. In the zoomed images, the arrows indicate the colocalization of GFP-LC3 puncta, lysosome, and Parkin in HCV-infected cells (white spots). (PDF) [file ppat.1003285.s010.pdf]

# Figure S11

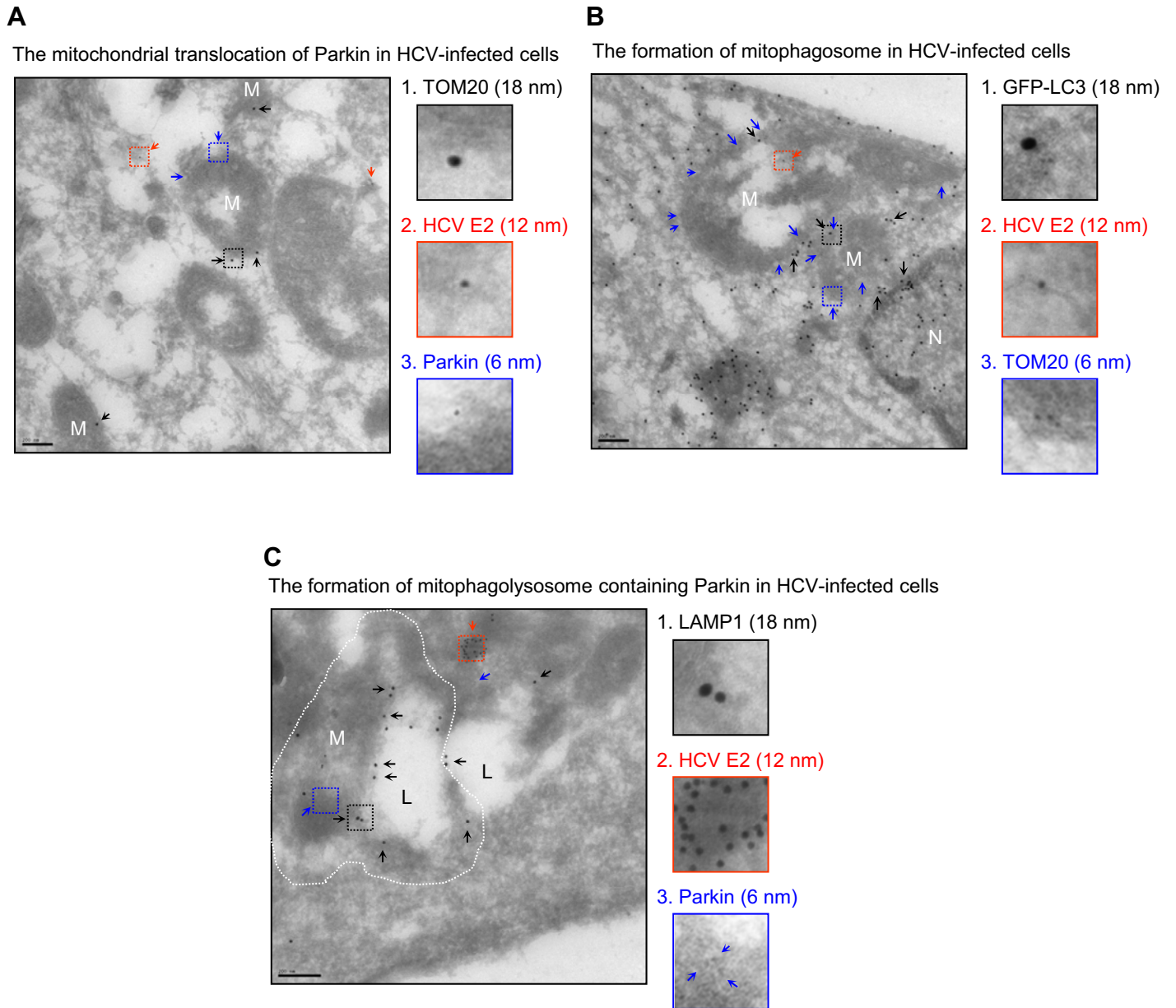

Supplement: Figure S11 — Immunoelectron microscopy of HCV infected cells. (A–C) Huh7 cells were infected with HCVcc. At 3 days post-infection, cells were fixed, processed for immuno-EM with the indicated antibodies, and examined by electron microscopy. (A) Ultrastructure of HCV-infected cells showing the mitochondrial translocation of Parkin. In the zoomed image, the gold particles indicate TOM20 (black arrow, 18 nm), HCV E2 (red arrow, 12 nm), and Parkin (blue arrow, 6 nm) in the damaged mitochondria with loss of mitochondrial cristae in HCV-infected cells. (B) Ultrastructure of HCV-infected cells showing the formation of mitophagosome. In the zoomed image, the gold particles indicate GFP-LC3 (black arrow, 18 nm), HCV E2 (red arrow, 12 nm), and TOM20 (blue arrow, 6 nm) in the damaged mitochondria of HCV-infected cells. (C) Ultrastructure of HCV-infected cells showing the formation of mitophagolysosome containing Parkin. In the zoomed image, the gold particles indicate LAMP1 (black arrow, 18 nm), HCV E2 (red arrow, 12 nm), and Parkin (blue arrow, 6 nm) in the fusion of damaged mitochondria-containing vesicles with the lysosome in HCV-infected cells. The mitophagolysosome formation is demarcated with white dot circle. Organelle marker: N, nucleus; M, mitochondria; L, lysosome. Scale bar = 200 nM. (PDF) [file ppat.1003285.s011.pdf]

**Figure S12**

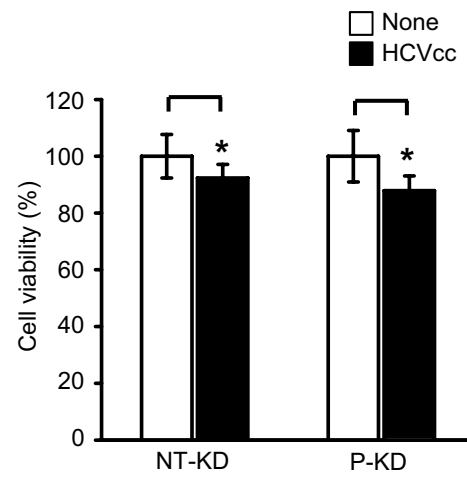

Supplement: Figure S12 — Viability of Parkin knockdown cells after infection with HCVcc. NT-KD and P-KD cells were infected with HCVcc. At 3 days post-infection, cell viability was measured as described in Materials and Methods. Cell viability is expressed as the percentage of the viable cells in the infected cells relative to the uninfected controls (mean ± SD; n = 3; *p<0.05). P values were calculated by using an unpaired Student's t-test. (PDF) [file ppat.1003285.s012.pdf]
