# Supplementary material for: Hepatitis C Virus Induces the Mitochondrial Translocation of Parkin and Subsequent Mitophagy
Source: PLoS Pathog. 2013 Mar 28;9(3):e1003285. doi: 10.1371/journal.ppat.1003285 (PMC3610669; doi:10.1371/journal.ppat.1003285)
Supplement: Figure S3 — HCV induces the mitochondrial translocation of Parkin in HCV full-length or subgenomic replicon-bearing cells. (A–D) Representative confocal images showing endogenous Parkin translocation to the mitochondrial perinuclear clusters in cells stably expressing HCV replicons. Stable cells harboring HCV full-length replicon FLR-JFH1 (genotype 2a), subgenomic replicon SGR-JFH1 (genotype 2a), and subgenomic replicon BM4–5 Feo (genotype 1b), respectively, and human hepatoma Huh7.5.1 cells were immunostained with anti-Parkin antibody. MitoTracker (Mito) was used for staining live mitochondria before fixation. The expression of HCV proteins (light gray) is verified by immunostaining with anti-HCV core (A) or NS5A antibody (B, C, and D). Nuclei were stained with DAPI (blue). In the zoomed images, yellow color indicates the colocalization of Parkin (green) with mitochondria (red). (E) ImageJ quantification of Parkin associated with mitochondria is described (mean ± SEM; n≥10 cells, *p<0.001). P values were calculated by using an unpaired Student's t-test. (PDF) [file ppat.1003285.s003.pdf]

Figure S3

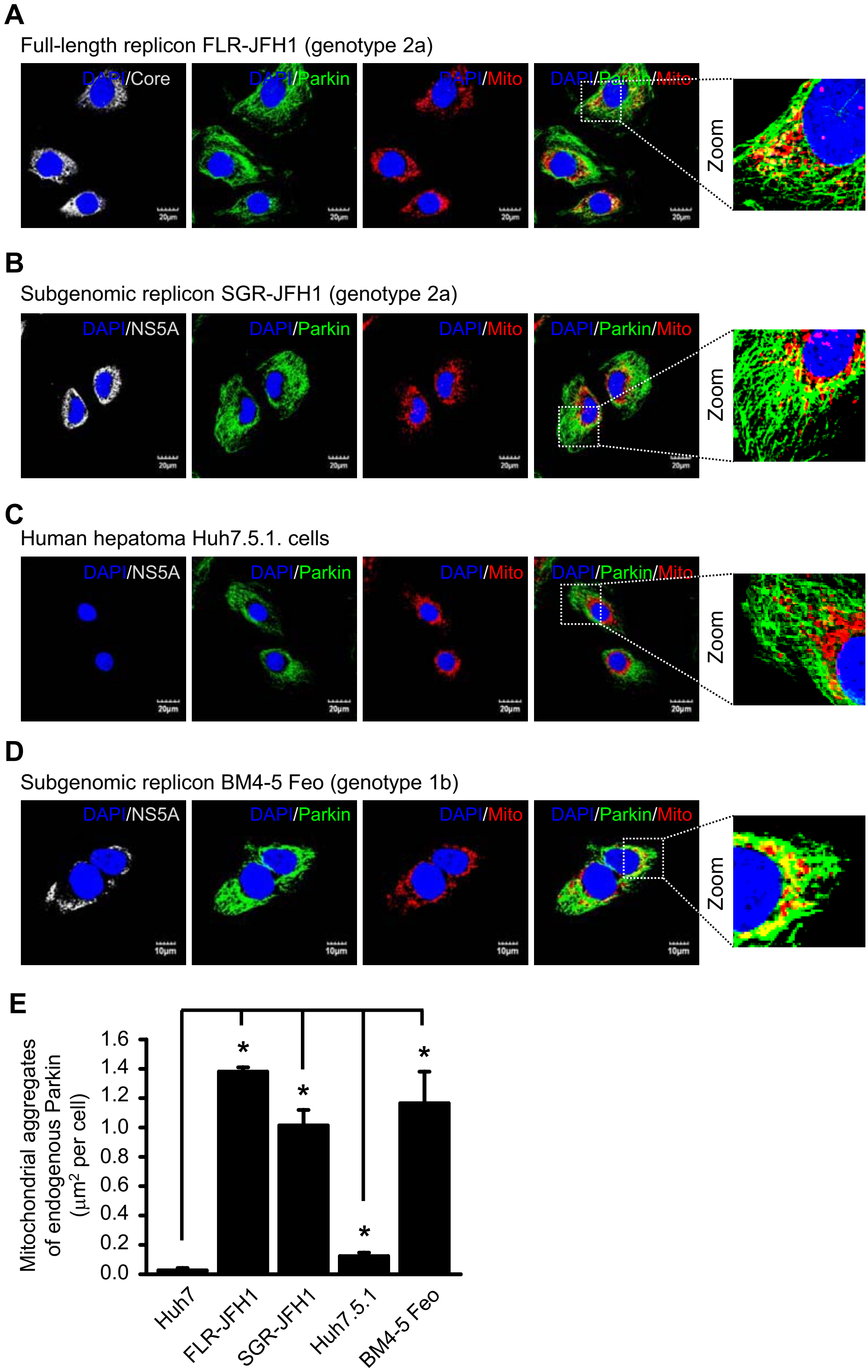
A
Full-length replicon FLR-JFH1 (genotype 2a)
DAPI/Core
DAPI/Parkin
DAPI/Mito
DAPI/Parkin/Mito
Zoom
B
Subgenomic replicon SGR-JFH1 (genotype 2a)
DAPI/NS5A
DAPI/Parkin
DAPI/Mito
DAPI/Parkin/Mito
Zoom
C
Human hepatoma Huh7.5.1. cells
DAPI/NS5A
DAPI/Parkin
DAPI/Mito
DAPI/Parkin/Mito
Zoom
D
Subgenomic replicon BM4-5 Feo (genotype 1b)
DAPI/NS5A
DAPI/Parkin
DAPI/Mito
DAPI/Parkin/Mito
Zoom
E
Mitochondrial aggregates of endogenous Parkin (µm² per cell)
1.6
1.4
1.2
1.0
0.8
0.6
0.4
0.2
0.0
Huh7
FLR-JFH1
SGR-JFH1
Huh7 5.1
BM4-5 Feo
